# Understanding the influence of power dynamics in intersectoral collaboration: A realist evaluation in Assam, India

**DOI:** 10.1371/journal.pgph.0005639

**Published:** 2025-12-12

**Authors:** Praveenkumar Aivalli, Brynne Gilmore, Prashanth Nuggehalli Srinivas, Aoife De Brún

**Affiliations:** 1 UCD Centre for Interdisciplinary Research Education and Innovation in Health Systems (UCD IRIS Centre), School of Nursing Midwifery and Health Systems, University College Dublin, Dublin, Ireland; 2 School of Nursing Midwifery and Health Systems, University College Dublin, Dublin, Ireland; 3 Center for Health Systemss, Institute of Public Health Bengaluru, Bengaluru, India; University of Washington Bothell, UNITED STATES OF AMERICA

## Abstract

Intersectoral Collaboration (ISC) involves multiple sectors working together to tackle complex challenges that no single sector can address alone. In global health, where interconnected issues demand holistic approaches, ISC aligns goals and resources to enhance effectiveness and equity. However, power dynamics within and between sectors can either foster synergy or create tensions, shaping ISC outcomes. This study explores how, why, for whom, in what contexts, and to what extent power dynamics influence ISC in a northeastern state of India. A realist evaluation was conducted in Assam, India. Six Programme Theories (PTs) from a prior realist review were tested and refined through theory-driven realist interviews with 18 stakeholders across different sectors. Data were analysed using Context-Mechanism-Outcome Configurations (CMOCs) to refine PTs, offering a nuanced understanding of how individual, institutional, and contextual factors influence ISC outcomes. A total of 62 CMOCs, grouped into 17 demi-regularities, refined six PTs on power dynamics in ISC. Fair participation fostered empowerment, while proactive leadership enhances motivation, though resource gaps may weaken these effects. Fair resource allocation reduced power imbalances, improving collaboration, whereas hierarchy and unclear roles breed distrust. Personal relationships helped build trust and overcome hierarchy. Findings emphasise that improving ISC requires attention to both structural and relational mechanisms through designing programs that leverage both of these. Given this single-district, qualitative case study, the findings are context-specific to Dibrugarh, Assam, and should be transferred cautiously to comparable settings. Future research could refine programme theories into a middle-range theory, enhancing their transferability to other settings.

## Background

As the importance of health system strengthening has been increasingly recognised globally, Intersectoral Collaboration (ISC) has emerged as a critical strategy to support this effort [1–3]. ISC involves the collaboration of various sectors, which combine their resources and expertise to enhance the health system’s capacity to effectively implement health programmes [4–6]. This integrated approach is essential because the success of health initiatives often depends on the active participation and cooperation of multiple sectors. Consequently, ISC has become a cornerstone in the broader strategy of strengthening health systems to meet global or national health objectives, ensuring that health systems are resilient and capable of addressing complex health challenges [7–12].

The World Health Organization (WHO) has also acknowledged the necessity of ISC in their statement on Intersectoral Action for Health, which calls for “a recognised relationship between part or parts of the health sector with parts of another sector which has been formed to take action on an issue to achieve health outcomes (or intermediate health outcomes) in a way that is more effective, efficient or sustainable than could be achieved by the health sector acting alone” [13]. In academic literature, various terms such as ‘intersectoral action,’ ‘multi-sectoral collaboration,’ ‘healthy public policies,’ and ‘cross-sector collaboration’ are often used interchangeably with ‘intersectoral collaboration’ [14–16]. However, the term ‘collaboration’ does not have a consistent definition or application across various disciplines, professions, and sectors. Moreover, there is a lack of research exploring the practical realities and practices of ISC in policymaking [17–19]. ISC is inherently complex, involving technical and political dimensions that require careful attention to power dynamics and inter-sectoral relationships [20]. The success of ISC initiatives in public policy and health systems depends significantly on how power is distributed and exercised among the collaborating sectors [4,21–23].

Power refers to the capacity or ability of an individual, group, or institution to influence or control the behaviour, actions, and decisions of others [24,25]. Power is not just about overt control; it can also be subtle, operating through norms, ideas, and the ability to shape perceptions and preferences [26,27]. In social and political contexts, power often involves the capacity to set agendas, frame issues, and determine which voices are heard or marginalised [28–30]. In the context of ISC, power is central to understanding how different stakeholders interact, make decisions, allocate resources and work towards a common goal. Power dynamics can shape the efficacy and outcomes of these collaborations, making it essential to understand and navigate these relationships carefully. For instance, power dynamics can significantly influence the effectiveness and equity of collaborative efforts, as those with more power can shape outcomes in ways that reflect their interests and priorities [31,32]. However, there remains a substantial gap in theoretical frameworks that capture how power operates within ISC. This gap highlights the need for more research to deepen our understanding of power’s role in shaping ISC outcomes, ensuring that collaborations are not only technically sound but also politically and socially equitable [33].

ISC in Low- and Middle-Income Countries (LMICs) poses distinct challenges and opportunities compared to high-income countries. A significant challenge is the limitation of resources [34–36]. The governance environments in LMICs often impede the effective management of ISC. Weak law enforcement, shifting policies, low transparency and accountability, and widespread corruption create major barriers to intersectoral action [37–39]. Compounding these structural constraints, the competitive push by funders and global health actors often disrupts local governance mechanisms and intersectoral relationships [40,41].

Many international agencies operate within rigid funding cycles and project-based mandates that prioritise short-term, measurable outcomes over sustained systemic change [42,43]. This competitive funding environment not only forces local institutions into donor-driven priorities but also fosters a culture of fragmentation, where ministries and local organisations compete for external resources rather than collaborate on long-term, integrated solutions [44]. Furthermore, Global actors sometimes distrust local structures, citing accountability and efficiency. This can sideline governance frameworks and relationships that are vital for intersectoral coordination [45]. This top-down imposition of priorities and decision-making authority often results in parallel systems of governance that weaken local ownership and long-term sustainability [46].

For example, a qualitative study conducted in Saharan Africa highlighted that policies with cross-sectoral aims often clash with existing resource allocation patterns, leading to competition among ministries [47]. Other research has emphasised that effective ISC in LMICs requires clear sectoral roles, strong leadership, accountability, governance, and a supportive political environment [47–51]. However, LMICs often struggle with weak institutions, limited funding, low salaries, poor enforcement of laws, and corruption, which impede ISC [39,52,53]. For example, in South Africa, a lack of supportive organisational structures undermined attempts at frontline collaboration for psycho-social rehabilitation [54]. Additionally, in Kenya, poor communication and unclear sectoral roles hindered collaborative efforts in health policies [55]. Overall, the evidence for ISC in LMICs tends to report failure rather than success, and there are few detailed and practical examples of successful ISC in practice.

Several countries have overcome barriers to implementing ISC by mainstreaming intersectoral approaches to health. A common theme in these successes is that the government, including the health sector, recognised the importance of intersectoral action. For instance, Iran established several national mechanisms for bringing sectors together to improve health, including the National Coordination Council for Healthy Cities and Healthy Villages [56]. The council looks after community-based health improvement initiatives based on strategies such as expanding access to financial credit, social services, and sanitation [57]. Vietnam has established a national intersectoral coordination mechanism, the National Traffic Safety Committee, with representatives from 15 ministries and agencies to advise the prime minister on improving road safety [58]. The committee was crucial in passing Vietnam’s national mandatory helmet law [58]. Conditional cash transfer programmes in Brazil provided an important opportunity for ministries of health to work with ministries of social development and others to care for the whole individual, which reduced under-five mortality rates [59–61]. These examples of successful ISC in action not only indicate how challenging it is to achieve but also demonstrate the potential of ISC. If ISC is formed and implemented appropriately, programmes can achieve better outcomes in a sustainable manner.

### ISC in India’s health and nutrition sector

ISC in India originates in early public health initiatives, notably influenced by the Alma-Ata Declaration of 1978, which highlighted the importance of primary health care and intersectoral action [62]. The National Rural Health Mission (NRHM), established in 2005, represented a significant advancement in strengthening ISC by aiming to improve access to quality health care through multi-sectoral engagement [63]. More recently, the National Nutrition Mission (POSHAN Abhiyaan), launched in 2018, was designed to address the challenge of malnutrition [64]. The programme encompasses six core components: Capacity Building, Intersectoral Convergence, IT-based data reporting, Innovations, and Behavioural Change and Communication (BCC) for nutrition. Of particular importance is the Intersectoral Convergence component, which serves as the backbone of the mission’s implementation. It establishes collaborative platforms at all administrative levels from the central to the village level. At state level, senior administrative heads chair convergence committees to coordinate efforts. At village level, VHSNDs provide a shared forum for health and social welfare sectors to work together.

### Operationalising ISC within the National Nutrition Mission

Operationalising ISC under the National Nutrition Mission (NNM) starts with identifying nutrition-specific and sensitive interventions across various departments. It involves state, district, block administrations, and local governments facilitating partnerships through established platforms. ‘Convergence Committees’ are tasked with creating annual ‘Convergence Action Plans’ that outline key nutrition actions for different departments. A nutrition framework has been developed to guide these committees, detailing indicators and targets for monitoring and evaluation. These committees review progress quarterly, identify gaps, and implement interventions to address them. Despite the emphasis on ISC and central guidance, the extent of synergistic effects from simultaneous sectoral collaboration to combat child undernutrition remains uncertain. In LMICs, efforts to reduce child undernutrition are often confined to health or women and child development departments, with limited integration with policy sectors that affect socio-economic and environmental conditions. Consequently, the potential benefits of ISC are not fully realised without empirical assessments of how different sectors can enhance action and share accountability [65,66].

Recent studies are bridging the theoretical gap by exploring power and influence in ISC through diverse approaches. A recent realist review has theorised and evaluated how power dynamics shape ISC, particularly in LMICs [67]. This review highlights power’s critical role in facilitating or hindering collaboration across sectors. Given this backdrop, examining power dynamics within ISC becomes imperative, particularly in large-scale health initiatives like India’s NNM. The present study was conducted to explore how, why, for whom, in what circumstances, and to what extent power dynamics promote or impede ISC within nutrition programming in Assam, India.

This study employed a Realist Evaluation (RE) approach [68], focusing on Context-Mechanism-Outcome (CMO) configurations through a case study methodology to offer nuanced insights into intersectoral power dynamics. Realist methods emphasise generative causation, where outcomes are produced through the interaction between mechanisms and context, as mechanisms are ‘triggered’ and operate differently depending on the specific contextual conditions [69,70]. This philosophical approach provides a distinctive perspective on empirical evaluation by recognising that both material and social worlds are real, with systems embedded within larger frameworks and interacting across levels [71]. Social systems, including programmes and policies, involve dynamic interactions between individuals, institutions, and broader social processes, resulting in various effects [72,73]. Realist epistemology acknowledges that there is no final truth, only ongoing improvement in understanding. Thus, RE does not seek absolute certainty but aims to enhance comprehension over time [73].

RE examines how mechanisms within intersectoral collaboration (ISC) produce varying outcomes depending on specific contexts and circumstances. These outcomes are shaped by a range of social, political, and theoretical factors [74–76]. In particular, studies have demonstrated that the success of an intervention is highly influenced by the context in which it is implemented [66,77,78]. Within realist approaches, context is understood as being shaped by the actions and interactions of individuals rather than merely external factors [79]. This highlights the importance of considering both contextual dynamics and actor-driven influences when evaluating the success of an intervention. RE is increasingly used to assess programmes and policies by investigating what works for whom under specific conditions, rather than simply evaluating overall efficacy [80–86]. It also explains how certain mechanisms work, why they work and how they produce specific effects [73,87–89]. This unpacking of generative causation provides an opportunity to understand complex causal relationships. Thus, the final explanation of a programme considers how and why programmes work (or fail to work) in specific contexts. These CMO configurations are then synthesised to develop broader theories that can be tested and refined [69]. Hence, the RE approach is well-suited to investigating how power dynamics within ISC produce different outcomes in varying contexts, identifying underlying mechanisms and their effectiveness [90].

### Aim

This research aims to improve the context-specific understanding of the influence of power in intersectoral collaboration within nutrition programming in Assam, India.

### Research question

How, why, for whom, in what circumstances and to what extent do power dynamics promote or impede intersectoral collaboration within nutrition programming in Assam, India?

## Methods

**Fig 1:**
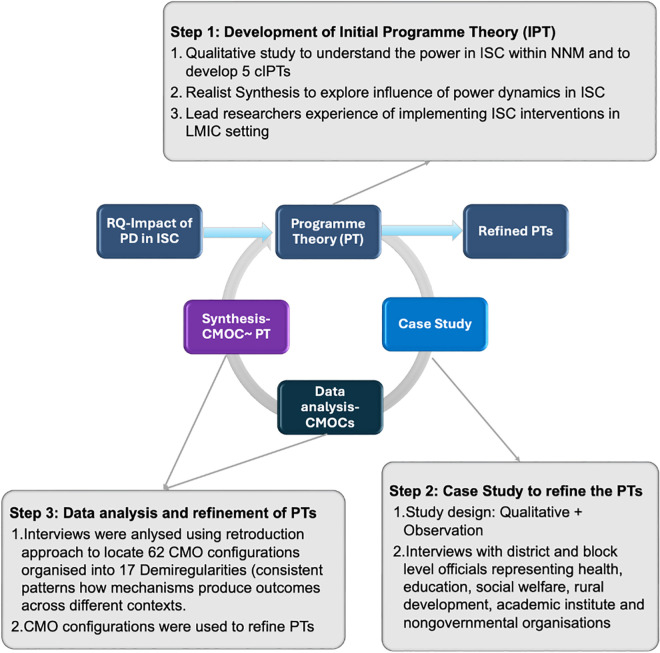
Realist evaluation framework and phases.

A realist evaluation was undertaken to address the research question. The design comprised three sequential steps, shown in Fig 1.

### Step 1: Development of initial programme theory

First step began with defining the research question, focusing on how power dynamics influence ISC outcomes. A qualitative study was conducted to understand implementors’ perspectives on ISC, which served as a basis for developing initial rough programme theories [91]. This was followed by searching existing literature and implementation experiences of the lead researcher, leading to the identification of five candidate Initial Programme Theories (cIPTs) – see S1 Text. A realist review was then conducted to refine these cIPTs by critically appraising and synthesising existing evidence to refine the cIPTs into Programme Theories (PTs). This realist review resulted in six PTs - see Table 1.

**Table 1:** Programme theories informed by realist synthesis [67].

IPT 1 Ownership:	During the policy formulation phase, granting equal ownership to different sectors or departments triggers increased stakeholder participation, coordination, and collaboration. This shift helps balance power dynamics during the planning and implementation of health interventions. By ensuring that all sectors share ownership, stakeholders feel more valued, recognised, and empowered. This sense of inclusion fosters deeper engagement and promotes a stronger commitment to collaborative intersectoral action, resulting in more cohesive and effective programme outcomes.
IPT 2 Leadership & goal alignment:	When leadership provides support for interventions and sector goals are aligned with sectoral priorities, coupled with adequate resources, it motivates and empowers staff. This alignment fosters a sense of team efficacy and shared responsibility, which strengthens engagement and accountability. The key mechanisms at play here are leadership backing and resource alignment, which drive staff motivation and connectedness to the broader system, thereby increasing the likelihood of successful intersectoral collaboration.
IPT 3 Resource:	When resources are unevenly distributed and inaccessible to certain sectors, it deepens dependency in weaker sectors and amplifies power imbalances. This dynamic weakens engagement and reduces collaboration during programme implementation. The primary mechanisms here are unequal resource allocation and increased dependency, which undermine collective action and diminish the effectiveness of intersectoral interventions.
IPT 4 Trust and hierarchies:	In hierarchical settings, if sectors’ views are not respected or information from one sector is distrusted by others, it discourages open communication and reduces participation. The key mechanisms here are lack of trust and perceived disrespect, which hinder coordination and collaboration, ultimately leading to poor engagement in intersectoral programmes.
IPT 5 Role clarity:	When role clarity is lacking in policies or guidelines, it creates confusion and conflict among sectors and implementation teams. The main mechanisms here are role ambiguity and miscommunication, which reduce engagement, efficiency, and accountability, ultimately straining interpersonal relationships and hindering effective implementation of programmes.

### Step 2: Case study and refinement of PTs

#### Setting.

A case study was conducted to test and refine the subsequent PTs providing contextual insights into the influence of power in ISC. Assam a state in northeast of India was the site for this study. Assam state has 33 districts with population typically between 2–3 million per district. For the current case study, Dibrugarh district was selected as it holds significant importance due to its strategic location in Upper Assam, serving as a major commercial hub and gateway to eastern parts of the state. The district is divided into seven blocks for administration purposes. It also has diverse ethnic communities and is prone to socio-political tensions, which can affect the implementation of public health initiatives [92]. The district encompasses an approximate geographical area of 2,000 square kilometres and has a population of 1.3 million [93]. The literacy rate in this district is between 65% and 70%, reflecting a moderate level of educational attainment among the population [94]. According to the National Family Health Survey - 5 conducted in 2019–2020, about 30% of children in Dibrugarh are stunted, indicating long-term nutritional deficiencies that affect growth and development. Wasting, which signifies acute malnutrition, affects 20% of children, while 30% are underweight, combining both chronic and acute malnutrition issues [95]. Furthermore, an alarming 80% of children under the age of five suffer from anaemia, highlighting significant iron deficiencies and other nutritional inadequacies. In the district, a District Nutrition Committee comprising various sectors as part of the Nutrition Mission has been constituted in early 2028-19 and is expected to convene monthly to discuss the progress of implementing nutrition interventions within the district.

#### Participants.

Key personnel involved in the implementation of the NNM through ISC, including district and block coordinators, development partners, and representatives from sectors such as health, education, and rural development, were interviewed to gather, qualitative data on the dynamics of ISC and the influence of power. Invitations were sent to the relevant organisations, requesting participation of key individuals from various sectors.

#### Data collection.

A realist interview study design was employed to collect data through the realist interview interviews, drawing on the methodology outlined by Ana Manzano [84]. In addition to interviews, observation data was also used. In realist evaluation, the evaluator (or interviewer) initially presents their theories or hypotheses to the participant, who then provides feedback, insights, or new information that helps to refine or challenge these theories. This process allows the participant to “teach” the evaluator about the realities and nuances of the context under investigation. The evaluator, in turn, refines their understanding and theoretical framework based on this exchange, making the participant a key source of knowledge in the realist evaluation process [96].

#### Interview guide.

The realist interview guides were developed to test and refine PTs that were developed based on the Realist Review, with questions focusing on key aspects from each PT. To assess ownership and empowerment, questions explored the effects of equitable ownership on engagement and collaboration. For leadership and goal alignment, questions examined the role of supportive leadership and resource alignment in motivating staff. Prompts on resource distribution addressed the impact of resource disparities on sectoral dependencies, while questions on trust and hierarchy explored the influence of respect and communication on intersectoral dynamics. Lastly, role clarity questions identified the effects of role ambiguity on programme efficiency. This guide provided a structured basis for testing and refining each PT in practice. Based on the first two interviews, slight modifications were made to the interview guide if required.

### Step 3: Data analysis

Aligned to realist evaluation methodology [97], data analysis was retroductive. Retroduction refers to “the identification of hidden causal forces that lie behind identified patterns or changes in those patterns” [98]. Retroduction employs both inductive and deductive reasoning, alongside researcher insights, to understand generative causation by exploring the underlying social and psychological factors identified as influencing programme outcomes. Following Maxwell’s realist approach [99] to categorising and connecting strategies for qualitative data analysis, the data from the single case study in Dibrugarh, comprising seven blocks, were first analysed block-wise and then compared across blocks to identify both converging and diverging patterns of evidence [100]. The analysis was conducted block-wise, with CMO coding managed through NVivo 11 [101].

The analysis proceeded in three phases. First, each transcript was reviewed for familiarisation. Second, an inductive coding round deepened engagement with the data and ensured attention to material beyond the a priori programme theories. Third, data were extracted as discrete CMOCs: each CMOC was written as an ‘if-then-because’ statement. The ‘if’ set out the context, the ‘then’ described the outcome, and the ‘because’ explained the mechanism. These were recorded in memos and coded to the node of the corresponding programme theory. Each programme theory node was linked to a memo that collated the relevant CMOCs, supporting excerpts, and observational notes indicating whether the evidence supported, challenged, or suggested refinement of the theory.

The synthesis drew on abductive and retroductive reasoning to refine programme theories and develop an account of generative causation. Retroduction was used to infer latent causal forces behind observed patterns and shifts in patterns, combining inductive reading, deductive checks, and the researcher’s analytic insight. In practical terms, each CMOC was printed on a separate slip of paper; these slips were moved on a desk to test links and sequences. This hands-on step made similarities and gaps clear. It also helped to identify when different CMOCs were versions of the same idea.

CMOCs were merged when the contexts matched in substance, the mechanisms operated in the same way, and the outcomes were functionally similar. Small wording differences were not a barrier. CMOCs were not merged when there was disconfirming evidence that showed a meaningful split. Every merge was logged in an audit trail with a short memo, at least one example, and a counter-example where available. The aim was to form one clear and useful CMOC rather than several near-duplicates. As this organising exercise repeated, recurring patterns became visible. These were treated as demi-regularities. A demi-regularity was defined as a partial pattern where a similar mechanism fired under comparable contexts and produced similar outcomes. Finally, CMOCs were grouped under their broad demi-regularities and linked back to programme theory nodes. Each group and each CMOC had a memo that recorded its label, defining context, mechanism family, typical outcomes, key quotes, boundary conditions, and any disconfirming cases. This produced a transparent chain from familiarisation, to CMOC drafting and observational confirmation, to merging, to demi-regularities, and then to programme theory refinement.

### Ethical statement

This study received ethical approval from two institutional review boards. Local ethical clearance was obtained from the Institutional Ethics Committee (Human), Assam Medical College, Dibrugarh, Assam, India (Approval No. 2023/AMC/EC/315E, dated 19 June 2023). Additionally, the study was reviewed and approved as a low-risk study by the Human Research Ethics Committee – Life Sciences (HREC-LS) at University College Dublin (UCD), Ireland, under reference number LS-LR-23–250.

All participants were provided with an information sheet outlining the study objectives, their rights as participants, and how data would be managed and protected. Written informed consent was obtained from all participants prior to the commencement of interviews. Participants were assured of the voluntary nature of participation, their right to withdraw at any point, and that all data would be anonymised to ensure confidentiality. Interviews were audio-recorded with permission, and data were securely stored and managed in accordance with UCD’s data protection policies and the EU General Data Protection Regulation (GDPR).

## Results

A total of 18 interviews with key stakeholders were conducted from December 2023 to April 2024. Interviews, approximately 40 minutes each. Table 2 provides details of the participants. Out of the 20 participants invited, 18 agreed to participate in the interviews.

**Table 2:** Distribution of key stakeholders interviewed across different sectors and administrative blocks in Dibrugarh district.

Sector	Block 1	Block 2	Block 3	Block 4	Block 5	Block 6	Block 7	Total Participants
Health	P1			P2		P3		3
Social Services	P4	P5		P6	P7			4
Education			P8	P9				2
Community		P10	P11		P12		P13	4
Public Administration	P14			P15				2
Non-Governmental Organisations						P16		1
Academia					P17			1
Development Partners	P18							1
**Total**	4	2	2	4	3	2	1	18

Participants were provided with an information sheet about the project and a consent form inviting them to participate voluntarily. Those who wished to participate returned the signed consent form, and an interview was arranged in person at a time and place convenient for them. The topic guide included questions related to their experience of the impact of power in initiating intersectoral partnerships as part of programme implementation, factors influencing sector partnerships on a shared goal, and drivers and barriers in establishing a good intersectoral partnership in the nutrition programme. Interviews were recorded, transcribed verbatim, and translated into English for analysis. Minor verbatim adjustments were made where required for clarity while preserving original meaning. Necessary permissions and approval were obtained from higher authorities (mission director) for this purpose. Additionally, snowball sampling was used to identify other key stakeholders.

A total of 103 CMOCs were developed from the 18 interview transcripts. These CMOCs were subsequently merged into 62 CMOCs based on similarity and pattern. A full list of 62 CMOCs is available in S2 Text. These 62 configurations were organised into 17 demi-regularities, each representing a recurring pattern observed across different contexts, mechanisms, and outcomes. Demi-regularities are partially consistent patterns of outcomes that occur when mechanisms (the reasoning/resources that drive change) interact with specific contextual factors (social, cultural, or structural conditions). They reflect the contingent nature of causality in social interventions, where outcomes depend on how, for whom, and under what circumstances a program works [85,102–104]. This process allowed for a systematic examination of how specific contexts and mechanisms interacted to produce particular outcomes. The analysis facilitated the identification of plausible patterns and contributed significantly to refining the PTs. Table 3 summarises the 17 demi-regularities along with their corresponding CMOCs and contributions to the overarching Programme Theories.

**Table 3:** PTs with corresponding demi-regularities, CMOCs, and sources.

PT	Demi-regularity	CMOCs
Inclusive Policy Development	Inclusive stakeholder engagement: Regular, inclusive meetings involving all relevant stakeholders to drive coordinated actions.	1, 2, 5, 7, and 10
	Community empowerment and influence: Recognising and harnessing the influence of community representatives in the decision-making process.	3, 4, 6, 8, 9 and 11
Leadership Support and Goal Alignment	Collaborative leadership and decision-making: Encouraging collaborative decision-making processes.	12, 13, 14, and 21
	Resource sharing and efficiency: Promoting equitable allocation of resources	16 and 18
	Structured and inclusive meetings: Conducting regular, structured meetings to address challenges and ensure inclusive decision-making.	15, 17, 19, 20, and 22
Resource Allocation	Balanced resource distribution: Ensuring fair and balanced distribution of resources to prevent dependency and foster collaboration.	23, 26 and 31
	External expertise and training: Leveraging external experts and providing extensive training for staff and community members.	24, 27 and 30
	Clear guidelines and resource management: Implementing clear policies for resource allocation and addressing resource constraints proactively.	25, 28, 29 and 32
Communication and Trust in Hierarchical Structures	Informal communication networks: Utilising informal channels, like WhatsApp, for streamlined coordination and engagement.	35, 37, 44, and 45
	Open and fair communication: Ensuring open communication and fair participation in meetings for collaborative decision-making.	34, 36, 39, 40, 41 and 42
	Building trust and compliance: Developing strong relationships to foster trust and ensuring compliance to streamline programme implementation.	33, 38 and 43
Role Clarity and Conflict Resolution	Role clarity and structured coordination: Ensuring well-defined roles and structured coordination mechanisms to enhance collaboration.	48, 49 and 50
	Managing influence and workload: Understanding department-specific influence and addressing staff workload for effective programme implementation.	47 and 49
	Effective attendance and knowledge gaps: Ensuring consistent participation and implementing strategies to bridge technical knowledge gaps.	46 and 51
Interpersonal Relationships	Building trust and collaborative leadership: Developing strong relationships to foster trust and encouraging collaborative decision-making.	53, 57, 58 and 62
	Relations beyond professional relationship: Regular, inclusive meetings involving all relevant stakeholders beyond professional boundaries	55, 56, 60 and 61
	Relations with community members: Recognising and harnessing the influence of community representatives in the decision-making process.	52, 54 and 59

### PT 1: Inclusive policy development and deployment

**Box 1. Presents the refined programme theory 1**

In hierarchical health system structures, when sectors are fairly involved in the policy formulation process and provided with equitable ownership, it fosters a sense of empowerment, trust, and commitment among participants. This reduces the dominance of more powerful sectors and mitigates dependency on specific departments, thereby limiting the influence of traditionally dominant actors. By ensuring that each sector’s perspectives are valued, acknowledged, and incorporated through structured mechanisms such as committees and task forces, this approach creates a more balanced environment where no single sector exerts undue control. The inclusion of both formal and informal channels of participation further promotes genuine engagement from all stakeholders, which helps to flatten hierarchical power dynamics. As a result, power is more evenly distributed, allowing for meaningful intersectoral collaboration, improved coordination, and enhanced overall effectiveness of interventions.

This PT suggests that fostering equal ownership in the policy development process enhances stakeholder participation, coordination, and collaboration. These dynamics, in turn, reduce dominance and dependency, leading to a more balanced and comprehensive approach to planning and implementing ISC interventions. This understanding emerged from a synthesis of 11 CMOCs developed through a combination of participant interviews and field observations. It was observed throughout this study that participants consistently emphasised the importance of government emphasis and priority in creating committees and task forces as central to ISC efforts. For example, a participant from the community sector remarked on the recent surge in such structures:

*Over the last couple of years, the government has placed significant emphasis on collaboration, forming committees and task forces. These committees have given us a platform to share our ideas and feel more involved in decisions.Early meetings felt repetitive. As convergence meetings became routine, all sectors started seeing the value of collaborative efforts. Discussions are now more aligned with action plans that include diverse perspectives.“(Participant 13).*

The quote reflects the government’s commitment to fostering collaboration through institutional power by regularising meetings and forming committees and task forces. Initially, these meetings were perceived as repetitive; however, over time, participants began recognising the value of coming together. This shift highlights how institutional power, when exercised positively, can cultivate inclusivity and camaraderie among stakeholders. The repeated convergence meetings not only helped align discussions with actionable plans but also fostered a sense of connectedness among participants. One participant highlighted how their involvement in task forces and being called to these meetings made them feel important and valued. Rather than feeling burdened by the frequency of meetings, they experienced a growing sense of inclusivity and engagement, affirming the positive impact of institutional power in creating a collaborative environment. Another participant shared a similar experience, describing regular meetings where health and nutrition service delivery topics were often addressed, along with action plans for which department was responsible for specific tasks:

*“I have been attending these meetings for the last three to four months regularly. The agenda is set by a chair who is a senior officers invite other departments for meetings. Since the letter comes from senior officer everyone turns up. Several health and nutrition service delivery-related topics are discussed along with action plans on who needs to do what. Due to frequent meetings and follow ups most of us know our focus areas” (Participant 11)*

The senior leader, as chair, uses their authority to set agendas and ensure attendance through formal directives. While hierarchy dictates participation, it fosters an environment for consistent discussions on health and nutrition. This use of soft power enables formal and informal interactions, building relationships over time. Frequent meetings normalise collaboration, clarify roles, and align responsibilities. Regular participation and the collaborative nature of these meetings contribute to a sense of equitable ownership across sectors, aligning with the theory that involving all stakeholders in decision-making can mitigate the dominance of particular sectors. Many participants observed that these discussions improved focus, accountability, and engagement, highlighting how hierarchical power, when channelled effectively, promotes collaboration and sustained engagement. However, field observations also revealed that while formal structures such as committees and task forces promote inclusive policy development, there are tensions between the theoretical ideals of ISC and its practical implementation. For instance, although participants like Participant 11 reported regular attendance and inclusive agendas, observations suggested that meetings often appeared highly procedural. This formality risked undermining genuine engagement and collaboration. This sentiment is echoed by a participant from the social welfare sector, who provided a more critical view:

*“These meetings are just for namesake is what I feel because the government is putting a lot of emphasis on the convergent meetings these days. A lot of times, they discuss the same topics, which sometimes undermines the collaborative effort. However, meetings have started to happen regularly, and all stakeholders turn up during meetings.” (Participant 5)*

Observations revealed that agenda-setting by senior officers reinforces power imbalances, restricting the flexibility and openness needed for dynamic ISC. This participant’s frustration highlights that genuine ISC requires active and meaningful engagement, which procedural rigidity can stifle. Reflecting on participant insights and field observations, this PT was refined to capture both the potential and limitations of current ISC practices. While participants valued inclusion in decision-making, others expressed concerns about the repetitive nature of meetings. These observations identified a pattern where inclusive structures exist, but power imbalances persist, as senior officers often control the agenda, potentially marginalising certain perspectives. Formal structures foster participation but do not necessarily ensure meaningful collaboration or equitable ownership. This PT was refined to acknowledge that inclusive policy development requires more than formal structures; it demands addressing power imbalances and fostering genuine engagement. Success hinges on moving beyond token participation toward active, equitable ownership of the decision-making process across sectors.. Table 4 details the PT refinement process using this PT as an exemplar. The refinement process for all other PTs (from PT 2–6) available in S3 Text**.**

**Table 4:** Programme Theory 1 refinement process.

**PT 1**	**Inclusive policy development:** When health policy development ensures equal ownership and participation across various sectors and departments, it promotes inclusivity, mutual understanding, open communication, shared decision-making, equal resource allocation, shared vision, empowerment, and motivation. All sectors feel acknowledged and valued, leading to increased and sustainable engagement.				
**CMOC** **No**	**Context**	**Mechanism**		**Outcome**	**Supporting quote(s)**
		**Resource**	**Reasoning**		
1	Emphasis on collaboration through structured committees and task forces in a hierarchical system.	Regular meetings and task forces provide **formal channels** for participation and decision-making.	Stakeholders feel **empowered and valued** as their input is actively sought and incorporated into discussions.	Increased **trust, empowerment, and collaboration** across sectors, reducing dependency on dominant actors.	*“Over the last couple of years, the government has placed significant emphasis on collaboration, forming committees and task forces. These committees have given us a platform to share our ideas and feel more involved in decisions.” (Participant 13)* ”*When these committees were first formed, I wasn’t sure if they would actually work. But now, with regular meetings, we’re finally seeing some collaboration. For example, in the last meeting, social services got to highlight the gaps in welfare schemes that were affecting nutrition outcomes. Our suggestions were not only heard but actually included in the final action plan. It feels good to know that our sector has a voice and that we’re contributing meaningfully to the process.”* ***(Participant 4)***
	CMOc9: Write out the actual CMOC here.				
2	Frequent meetings led by senior officials but designed to include inputs from all sectors.	Structured **agendas and follow-ups** ensure clear roles and responsibilities for all stakeholders.	Stakeholders experience **role clarity** and feel their contributions are essential to the programme’s success.	Enhanced **coordination and shared ownership**, fostering a balanced environment.	***“Frequent meetings with clearly defined roles and follow-ups have helped clarify our responsibilities and fostered mutual respect among departments. This makes collaboration more meaningful.”*** *(Participant 11)* *”These committees have given us a space to talk about the real problems in our villages. Before, it always felt like decisions were made far away, without any input from the community. But now, we’re invited to these meetings, and they ask for our perspectives. Last time, I raised concerns about malnourished children not getting regular Anganwadi rations, and the issue was taken seriously. It’s empowering to know that what we say actually matters.” (Participant 4)*
3	Convergence meetings align multiple sectors to jointly plan and execute health initiatives.	Government provides **inclusive platforms** for collaboration, integrating inputs from various stakeholders.	Stakeholders perceive **genuine engagement** and appreciate that their perspectives are valued equally.	Improved **coordination, reduced power imbalances**, and meaningful intersectoral collaboration.	***“Regular convergence meetings have improved participation from all stakeholders, and the inclusion of every department’s input has created a sense of shared ownership.” (Participant 5)***
4	Convergence meetings are regular but discussions risk becoming repetitive.	Government **prioritisation of collaboration** ensures frequent meetings to address collective goals.	Stakeholders feel **engaged but seek variety** in the topics discussed to remain fully committed to the process.	Reduced **repetition in discussions** and enhanced relevance of collaborative efforts.	***“Initially, meetings felt repetitive, but as convergence meetings became regular, all sectors started seeing the value of collaborative efforts. Discussions are now more aligned with action plans that include diverse perspectives.” (Participant 13)*** *At first, these convergence meetings felt like we were just going over the same points again and again, and it was hard to stay engaged. But as they became more structured, things started changing. In our last meeting, we discussed school health interventions, and for the first time, it felt like every sector brought something meaningful to the table. The discussions were focused on action—like aligning health check-ups with mid-day meal programmes—and it finally feels like we’re making real progress together.” – Participant 8*
5	Collaborative planning sessions where each sector has equal opportunity to highlight resource needs.	Structured forums with time allocated for all sectors to share priorities and gaps.	Inclusive discussions create a sense of ownership and respect, reducing tensions related to resource imbalances.	Strengthened collaboration and more equitable resource allocation decisions.	*“You know, earlier, planning meetings used to feel like a one-sided affair. Health would present its requirements first, and before anyone else could properly put their points across, the budget would already be allocated. Other dept like social welfare, NGO and ICDS barely got a chance to make their case. It wasn’t intentional, but it was happening, and it created tension. – Participant 11* *Now, things are different. DSWO ensures that every department gets equal time to talk about what they need. Last time, WASH spoke about how sanitation issues were affecting nutrition outcomes, and instead of brushing it off, we actually had a serious discussion about re-allocating some funds to fix it. It felt like real teamwork for the first time. When everyone gets a say, the decisions are stronger, and the results are better.“- Participant 13* *”Earlier, planning meetings were full of unspoken tensions. The departments with more power, more funding, and more say would dominate, and the others would just go along with it. It wasn’t fair, and it definitely wasn’t effective. But the way we do things now has changed the game. Participant 1* *Now, everyone gets structured time to speak. It sounds like a small thing, but it makes a huge difference. When we went sector by sector in the last meeting, we actually identified a major gap, funds were being over-allocated to outreach activities while school-based interventions were underfunded. Because we had open discussions, we were able to shift things around to balance it better. That’s the power of fair discussions, everyone gets what they need, not just what the powerful sectors decide.“Participant 1*
6	Convergence meetings align multiple sectors to jointly plan and execute health initiatives.	Government provides **inclusive platforms** for collaboration, integrating inputs from various stakeholders.	Stakeholders perceive **genuine engagement** and appreciate that their perspectives are valued equally.	Improved **coordination, reduced power imbalances**, and meaningful intersectoral collaboration.	***“Regular convergence meetings have improved participation from all stakeholders, and the inclusion of every department’s input has created a sense of shared ownership.” (Participant 5)***
7	Collaborative planning sessions where each sector has equal opportunity to highlight resource needs.	Structured forums with time allocated for all sectors to share priorities and gaps.	Inclusive discussions create a sense of ownership and respect, reducing tensions related to resource imbalances.	Strengthened collaboration and more equitable resource allocation decisions.	*Earlier, planning meetings felt like a race, with the bigger sectors dominating the conversation and smaller ones like ours barely getting a chance to speak. Now, the leadership ensures every sector has equal time to present their challenges. Last month, we highlighted gaps in social protection schemes for malnourished families, and instead of being overlooked, it was addressed right there in the meeting. It feels fair now, decisions are based on actual needs, not just who has more power* ***.“ – Participant 7***
8	Transparent reporting of resource usage by all sectors, monitored by a neutral third party.	Regular reporting frameworks and independent monitoring teams to review fund utilisation.	Transparency builds trust, ensuring all sectors feel accountable and valued in the collaboration process.	Improved trust and reduced conflicts, fostering a more egalitarian environment for intersectoral work.	*“In the past, there was always suspicion about how resources were being used, whether one department was taking more than its fair share. But now, with a neutral monitoring team reviewing fund utilisation, everyone feels more secure. We know that every sector is being held accountable, and that builds trust. Last time, when there were concerns about overlapping expenditures, the monitoring team stepped in and resolved it fairly. It’s refreshing to see such transparency.”* ***– Participant 18***
9	Smaller sectors are provided with dedicated budgets for capacity-building initiatives.	Training programmes and skill development opportunities for under-resourced departments.	Capacity-building boosts confidence and reduces dependency, enabling these sectors to contribute more actively	Enhanced self-reliance and stronger collaboration in intersectoral interventions.	*“Earlier, we always had to depend on the health department for guidance because we didn’t have the skills or training to handle certain responsibilities on our own. But with the new budget set aside for training, our team has learned how to manage nutrition-focused outreach programmes independently. Last month, we organised a community nutrition drive without needing help from other sectors. It feels good to finally take the lead and show that we’re just as capable.”* ***– Participant 6***
10	Policy drafts are co-signed by all sectors.	Joint accountability agreements.	Collective ownership fosters pride in outcomes.	High adherence to policy implementation.	*“When we used to get policies, it always felt like they were just handed down to us, with no real say in shaping them. But this time, when we saw all our department logos side by side on the final POSHAN Maah action plan, it felt different, like we all had a stake in making it work. It wasn’t just a health or ICDS document anymore; it was ours. That sense of ownership has made everyone more committed to actually implementing it.” – Participant 7*
11	Needs-assessment tools allocate resources.	Data-driven allocation algorithms.	Reduces subjective bias in funding.	Perceived fairness across sectors.	*“Before, funding decisions felt like they were based more on who had the right connections rather than actual need. But now, with data-driven tools, there’s no room for favoritism. Last time, when we allocated nutrition funds, we used real malnutrition data instead of just relying on department preferences. It was the first time I saw every sector accept the decision without any complaints, because the numbers made it fair.”* ***– Participant 15*** *”I can’t tell you how many times we’ve lost out on funding simply because bigger sectors had more influence in decision-making because of their political connections. But now, with needs-assessment tools, the process is transparent. During the last budget cycle, our request for additional staff got approved, not because we pushed for it, but because the data showed the gap clearly..”* ***– Participant 5***
Thought process	The PT highlights the broad concept of inclusivity in health policy development, focusing on equal ownership and participation across sectors. It identifies general mechanisms like mutual understanding, empowerment, and shared vision as drivers for sustainable engagement. However, it lacks specificity in terms of **how inclusivity functions in a hierarchical health system**, where power imbalances are common. The refined PT addresses this by situating the theory within the **hierarchical health system context** and introducing mechanisms like equitable ownership and structured participation through committees and task forces. This contextualisation makes the PT more actionable and relevant. • **Key Change to the PT:** The refined PT now narrows the focus to hierarchical health systems and explicitly acknowledges **power dynamics**, which were not emphasised in the earlier version of PT. It explains how inclusivity reduces dependency on dominant actors and fosters a balanced environment. The PT briefly mentions inclusivity and shared decision-making but doesn’t delve into the **power imbalances** that exist in many health systems. The refined PT incorporates a nuanced understanding of how equitable ownership and participation can help mitigate power asymmetries. Drawing on evidence from the CMOCs and participant quotes, it explains how structured mechanisms (like committees) can reduce the influence of powerful actors while empowering less dominant sectors. • **Key Change to the PT:** The refined PT now introduces **power redistribution** as a central concept. It explicitly connects **fair involvement in policy formulation** to reducing dependency on traditionally dominant sectors, an insight derived from CMOC discussions. The PT emphasises inclusivity and shared ownership but doesn’t specify the channels through which this inclusivity is operationalised. The refined PT integrates evidence from participant quotes and CMOCs to highlight the role of both **formal mechanisms (e.g., structured committees and task forces)** and **informal mechanisms (e.g., interpersonal communication and mutual trust)**. For instance, participant quotes illustrate how frequent meetings and clear agendas provide structure, while informal channels like trust and relationships enhance engagement. • **Key Change to the PT:** The refined PT now includes **formal and informal channels of participation**, recognising their combined role in fostering genuine engagement and trust. The PT touches on outcomes like motivation and sustainable engagement but doesn’t establish clear causal links between mechanisms (e.g., shared vision) and outcomes (e.g., sustained collaboration). The refined PT strengthens these links by using CMOC evidence to show how **empowerment, trust, and mutual respect** lead to **more effective collaboration and improved interventions**. For example, the CMOCs highlighted how structured agendas and task forces reduce confusion and ensure all stakeholders feel valued. • **Key Change made to the PT:** The refined PT now spells out how mechanisms such as shared ownership lead to outcomes like distributed power and stronger collaboration.. The PT uses aspirational language such as “mutual understanding” and “motivation”, which are important but somewhat abstract. The refined PT adopts a more **analytical and precise tone**, reflecting the complexities of hierarchical systems. It avoids overly optimistic terms and instead focuses on **practical changes** (e.g., reducing dominance, fostering equitable ownership) supported by the evidence. • **Key Change to the PT:** The refined PT is now more focused, analytical, and grounded in realist principles, ensuring it aligns with the observed data and quotes. Summary: The refinement process involved a critical review of the PT through the lens of the CMOCs and participant quotes. It considered the **context of hierarchical health systems**, identified key mechanisms that promote inclusivity, and clarified the causal pathways leading to positive outcomes. The refined PT is evidence-based and acknowledges the dual importance of formal structures and informal dynamics in achieving meaningful intersectoral collaboration. • **Evidence from Quotes:** Participant quotes such as **“frequent meetings with clearly defined roles have fostered mutual respect”** and **“committees have given us a platform to share ideas”** informed the inclusion of structured mechanisms and highlighted their impact on power redistribution and trust-building. • **Focus on Realism:** The refined PT addresses practical challenges (e.g., dependency on dominant sectors) while providing realistic pathways (e.g., reducing dominance through equitable involvement).				
Mechanism Elaboration: Triggers	**Key Components Added to refine PT2** PT1 now specifies **four sub-mechanisms** derived from participant data • **Structured Participation** ◦ *Trigger*: Mandated forums (e.g., monthly convergence meetings) with predefined agendas and rotating sector leadership. ◦ *Participant Evidence*: *“Regular convergence meetings have improved participation from all stakeholders... Discussions are now aligned with action plans.”* (Participant 13) • **Recognition of Sectoral Expertise** ◦ *Trigger*: Explicit inclusion of sector-specific priorities in policy drafts (e.g., education inputs in health initiatives). ◦ *Participant Evidence*: *“The inclusion of every department’s input has created a sense of shared ownership.”* (Participant 13) • **Role Clarity and Accountability** ◦ *Trigger*: Publicly documented action plans with assigned responsibilities and deadlines. ◦ *Participant Evidence*: *“Frequent meetings with clearly defined roles and follow-ups have helped clarify responsibilities.”* (Participant 13) • **Iterative Feedback Loops** ◦ *Trigger*: Post-meeting evaluations to refine processes (e.g., surveys on perceived inclusivity). *Participant Evidence*: *“Initially, meetings felt repetitive, but as they became regular, sectors saw the value.”* (Participant 13)				
Boundary conditions	PT1’s success hinges on **four contextual factors**: 1. **Cultural Acceptance of Inclusivity**: Stakeholders must view participatory norms as legitimate (not imposed). 2. **Resource Adequacy**: Forums require dedicated funding (e.g., logistics, secretariat support). 3. **Power Redistribution Incentives**: Dominant sectors (e.g., health) must cede agenda-setting space. 4. **Time for implementation**: Trust and shared ownership develop gradually (Participant 13: *“This change is gradual…”*).				
Temporal dimension	PT1’s outcomes unfold in **three phases**: 1. **Resistance Phase**: Scepticism due to legacy of tokenism (*“meetings felt repetitive”*). 2. **Trust-Building Phase**: Consistent mechanisms (e.g., role clarity, feedback loops) reduce ambiguity. 3. **Institutionalisation Phase**: Collaboration becomes self-sustaining (*“shared ownership”*).				
Refined PT 1	In hierarchical health system structures, when sectors are fairly involved in the **policy formulation process** and provided with **equitable ownership**, it fosters a sense of **empowerment**, **trust**, and **commitment** among participants. This reduces the dominance of more powerful sectors and mitigates dependency on specific departments, thereby limiting the influence of traditionally dominant actors. By ensuring that each sector’s **perspectives are valued**, **acknowledged**, and **incorporated** through structured mechanisms such as **committees and task forces**, this approach creates a more balanced environment where no single sector exerts undue control. The inclusion of both **formal and informal channels** of participation further promotes **genuine engagement** from all stakeholders, which helps to flatten hierarchical power dynamics. As a result, power is more evenly distributed, allowing for meaningful intersectoral collaboration, improved coordination, and enhanced overall effectiveness of interventions.				
*PT 1 Simplified*	**Refined Context-Mechanism-Outcome (CMO) Statement** *In hierarchical, resource-constrained governance systems where intersectoral collaboration (ISC) has historically been symbolic or siloed (Context), the institutionalisation of* ***structured participatory processes****—characterised by equitable representation, iterative feedback loops, transparent role allocation, and binding resource commitments (Mechanisms)—activates recognition of sectoral expertise, redistributes decision-making power, and fosters* ***shared ownership***, ***sustained engagement****, and* ***equitable implementation*** *of policies (Outcomes).*				
Policy Implications	To operationalise PT1: • **Embed Inclusivity in Governance Protocols**: ◦ Require cross-sector sign-offs on policy drafts (e.g., education, sanitation, health). • **Create Technical Secretariats**: ◦ Dedicated teams to manage meeting logistics, document decisions, and track follow-ups. • **Integrate Equity Audits**: ◦ Annual reviews assessing whether marginalised sectors (e.g., rural development) influenced decisions.				

### PT 2: Supportive leadership structures

**Box 2. Refined PT 2**

In hierarchical health system structures, when leadership actively supports open communication, aligns sector goals with sectoral priorities, and ensures appropriate resource allocation, it creates a supportive environment that motivates, empowers, and engages staff. This leadership-driven approach fosters team efficacy and shared responsibility, as staff members feel valued, supported, and confident in their ability to contribute meaningfully. Empowered by leadership, staff are more likely to take ownership of their roles, contribute proactively, and trust in their team’s collective capabilities. Transparent communication structures help break down hierarchical barriers, reduce power imbalances, and foster a sense of equity within the team. By creating a culture of openness and inclusivity, leadership promotes a collaborative environment where power dynamics are minimised, and staff are encouraged to work together towards common goals.

This PT suggests that leadership support, goal alignment with sectoral priorities, and appropriate resource allocation empower and engage staff, fostering team efficacy and shared accountability. In turn, these dynamics are expected to strengthen connections with the broader system, leading to improved engagement and collaboration in intersectoral interventions, while reducing dominance, hierarchical influence, and power imbalances. The theory is supported by 11 CMOCs derived from participants’ interviews and field observations during the study. It was observed throughout this study that participants frequently highlighted the critical role of leadership in clarifying roles, encouraging open communication, and facilitating collaborative efforts. For example, a participant from the community sector shared how supportive leadership was instrumental in creating a more collaborative environment:

*“The new senior officer has been a guiding force, not only clarifying our roles and responsibilities but also championing a good communication in ways that make collaboration feel essential, not optional. She brings departments together, reminding everyone that real progress only happens when we work as one. Always just a WhatsApp message away, her responses come quickly, making her support feel like a steady presence, even in the busiest moments.” (Participant 13)*

Participant 13’s account demonstrates how leadership support fosters a more inclusive and collaborative environment. Despite holding a senior position, the officer’s accessibility through informal channels, coupled with prompt communication, helps reduce hierarchical structures and minimises the prominence of power dynamics. This was reinforced by field observations, where it became apparent that this senior officer’s involvement was critical in promoting collective action. By modelling inclusive behaviours, the leader ensured that various sectors worked towards a shared goal, creating a sense of team efficacy and joint accountability. These observations, combined with participants’ views, led to the identification of a pattern suggesting that supportive leadership structures contribute to a coordinated environment, enabling sectors to work collaboratively towards shared objectives while diminishing hierarchical dominance. These findings were further reinforced by the reflections of Participant 12 from the community sector, who observed a substantial transformation in ISC meetings over the past year and a half. What were initially perceived as superficial formalities had evolved into forums for meaningful discussion and problem-solving. Participant 12 attributed this change to shifts in leadership and attitudes among the line departments involved in ISC:

*“Earlier, meetings seemed only for the namesake, but over the past one and a half years, there’s been a noticeable shift towards meaningful discussions and problem-solving. This change is gradual and stems from a change in leadership and the attitudes of the line departments involved in ISC. I believe that with consistent follow-up and concrete actions, this shift becomes ingrained.” (Participant 12)*

This account illustrates a significant shift from procedural, symbolic meetings to spaces for genuine dialogue and collective problem-solving. The participant attributed this transformation to changes in leadership and improvements in the attitudes of the involved sectors. This observation reflects the durability of leadership-driven changes that foster open discussion and shared responsibility, reducing dependence on individual leaders and mitigating hierarchical dominance.

These examples, combined with field observations, indicate that supportive leadership works contextually by leveraging the dynamics of a hierarchical health system which produces diminished hierarchical dominance and enhanced collaboration by engaging the key contextual factors that influence staff behaviour and organisational culture (see 2).

### PT 3: appropriate and equitable resource allocation

**Box 3. Refined PT 3**

In a low-resource setting, when resources are distributed equitably and made accessible to all sectors, it mitigates the dependency of less resource-endowed sectors on more powerful ones. This equitable distribution fosters a sense of fairness and inclusivity among stakeholders, empowering all sectors to participate more actively and meaningfully in collaborative processes. By reducing disparities in resource allocation, participants from less powerful sectors feel valued and capable, which fosters autonomy and diminishes the need to rely on dominant sectors. As sectors become more self-reliant, this balanced distribution of resources promotes better engagement, stronger collaboration, and more equitable decision-making. This dynamic reduces the influence of traditional power hierarchies in intersectoral interventions and creates a more egalitarian environment where mutual support is encouraged, ultimately enhancing the effectiveness of collaborative efforts.

This programme theory posits that equitable and appropriate distribution of resources among sectors mitigates the dependency of less influential sectors and addresses perceived power imbalances, thereby fostering enhanced sectoral engagement and collaborative action. The theory was supported by 10 CMOCs developed from both participant interviews and field observations. Participants frequently emphasised that equitable resource allocation was crucial in balancing power dynamics across sectors, allowing for more effective ISC. For example, Participant 8 from the education sector observed how variations in budget allocation and staff presence can affect sectoral power and influence:

*“Each sector possesses its own strengths and weaknesses. For example, when comparing budget allocation, the Rural Livelihood Mission tends to receive more funding compared to other sectors, with health following closely behind. The Rural Livelihood Mission also has a larger staff presence, as it focuses on empowering women through small-scale entrepreneurship initiatives. While the health sector may have a slight advantage in terms of qualified personnel, its budget allocation ranks second to the livelihood sector.” (Participant 8)*

Participant 8’s account highlights the differing capacities and resources across sectors, such as the substantial funding and staff presence of the Rural Livelihood Mission, which can create imbalances in power and effectiveness within ISC efforts. Observations during this study supported these views, revealing that the Rural Livelihood Mission, with its considerable resources, holds a significant advantage, potentially overshadowing the contributions of other sectors like health. From both participants’ perspectives and field observations, it became evident that recognising and strategically leveraging these sectoral strengths through equitable resource allocation could enhance overall collaborative efforts. Participants from the health sector also underscored the importance of resource sharing and cross-sector collaboration. Participant 3 provided a practical example of how the Rural Livelihood Mission’s community meeting platforms facilitated ISC by enabling women-centric awareness campaigns. They described how these platforms were repurposed to address broader health concerns, particularly around nutrition:

*“It’s interesting to note that the livelihood mission in my block provides us with great community meeting platforms. Our field staff often organise women-centric awareness campaigns there, and they even invite our experts for sessions sometimes, which is really encouraging to see. Typically, these meetings revolve around utilising untied funds for the betterment of women. What I proposed is that the same funds can also be used to address nutritional deficiencies in children at the household level. It was my responsibility to push this point because that’s how we can reduce the burden. In my block, a few livelihood associations have already started using untied funds for their children’s nutrition as well. Isn’t it motivating? Our Chief Executive Officer sir has been supportive of this initiative, which is fantastic. I believe that resource sharing is crucial if we want to see positive outcomes, regardless of the department/sector involved.” (Participant 3)*

Participant 3’s narrative illustrates how resources provided by the Rural Livelihood Mission, originally intended for women’s empowerment initiatives, were extended to address nutritional deficiencies in children, thanks to proactive advocacy. The use of untied funds across sectors not only facilitated ISC but also reduced dependency on any single sector. Leadership support, in this case from the Chief Executive Officer, was identified as instrumental in enabling this cross-sectoral resource sharing. It became clear that advocacy, combined with supportive leadership, could leverage available resources to push for initiatives that transcended traditional departmental boundaries.

Field observations further corroborated these insights. As resources became more evenly shared, previously marginalised sectors began to feel more valued and capable, which in turn increased their participation. This shift in resource control weakened the traditional power hierarchies, as no single sector could dominate the decision-making process purely based on resource advantage. The power imbalances were mitigated as sectors felt more empowered, leading to a more equitable, inclusive environment for collaboration. By leveraging the unique strengths of all sectors, ISC processes became more balanced, and the proactive resource sharing reduced the influence that historically dominant sectors wielded. These findings highlight the generative causation at play, demonstrating how fair allocation of resources in a resource-limited setting leads to a shift in power dynamics - from one characterised by dependency and hierarchy to one of shared responsibility and mutual engagement (see Box 3).

### PT 4: Hierarchical structures and trust

**Box 4. Refined PT 4**

In settings characterised by hierarchical structures, stakeholders may be hesitant to share their perspectives openly during ISC meetings if they feel that their views are not respected or that their contributions are undervalued by other sectors. This perceived lack of respect or trust creates psychological barriers, leading individuals to withdraw from discussions or withhold critical information. As a result, more powerful sectors or individuals dominate the decision-making process, exacerbating the hierarchical nature of the collaboration. This mechanism leads to the creation of an unseen power structure, where decisions become skewed in favour of those who hold more influence or authority. When participants do not feel heard or valued, the existing power imbalances within the hierarchy are reinforced, further undermining collaborative efforts and preventing the full participation needed for effective ISC.

This PT suggests that in hierarchical settings, when stakeholders perceive a lack of respect for their perspectives or distrust information from other sectors, they tend to withhold input during meetings. This lack of open communication hinders participation, coordination, and collaboration, resulting in poor engagement, limited information sharing, and reduced collaborative action within programmes. Insights from participants strongly supported this theory, which was underpinned by 13 CMOCs derived from both interviews and field observations. Participant 14 from the public administration sector highlighted the challenges hierarchical structures present in collaborative meetings which explains how hierarchical dominance can shape meeting dynamics and decision-making processes,

*“In meetings, it’s a cacophony of voices - everyone talks, but few truly listen. The senior officer from the health department takes the floor more often, wielding a wealth of information that subtly commands attention. His words carry weight, and, more often than not, it’s his points that make it into the official record. The health department’s influence looms large, shaping decisions in its favour while others struggle to break*

Observations during the study confirmed that the health sector, often perceived as superior due to the qualifications and professional status of medical personnel, tends to dominate discussions. This dominance creates an imbalance, where decisions are disproportionately influenced by the health department’s perspectives, often overshadowing or undervaluing input from other sectors. As a result, the diversity of contributions necessary for balanced and effective outcomes is limited. The unspoken dominance of one sector, as observed, highlights the challenges that hierarchical structures present in fostering truly collaborative intersectoral efforts. In response to these imbalances, some participants suggested practical ways to mitigate the communication challenges posed by hierarchical structures. Participant 5, for example, emphasised the importance of having predetermined discussion topics that incorporate input from all relevant departments to ensure comprehensive and inclusive dialogue:

*“In convergence meetings, the topics of discussions should be fixed, incorporating criteria from all the departments. All departments should be aware of each other’s indicators; if this happens from the state level, it would be much better.” (Participant 5)*

Participant 5’s suggestion emphasises the need for a more coordinated approach to meeting agendas, where each department’s key performance indicators and priorities are transparent and embedded into the discussion. As observed during the study, this approach could lead to more cohesive and informed decision-making processes at different hierarchical levels. By ensuring that all sectors are equally represented and their performance indicators are considered, the dominance of one sector can be reduced, and a more balanced ISC can emerge. Moreover, the necessity for broader inclusivity in meetings was advocated by Participant 16 from the NGO sector, who stressed the importance of equitable participation from all sectors and extending representation beyond traditional public members:

*“There should be fair participation from all the sectors. Meetings should also involve women members of the community, not just public representative members. As a public representative member, I don’t have much authority within the existing framework, I can only push things up to some extent.” (Participant 16)*

Participant 16’s perspective highlights the critical importance of inclusive participation, particularly involving community members to ensure diverse voices are heard. Observations during fieldwork revealed that public representatives, while playing a vital role, often face structural barriers and limited authority in decision-making, reinforcing the dominance of more powerful sectors. Participant 16’s comment draws attention to the limitations imposed by hierarchical structures and the need for a more inclusive approach that involves a wider range of stakeholders. Expanding participation beyond traditional role involvement can help mitigate these limitations and foster greater trust and collaboration among sectors. Reflective analysis of participant insights and field observations refined the understanding of how hierarchical structures and communication breakdowns influence trust and collaboration within intersectoral settings. The unbalanced power dynamics created by hierarchical dominance limit effective participation and reduce trust between sectors. Trust can be undermined when stakeholders feel their perspectives are not respected or when certain sectors dominate discussions, which leads to disengagement and reduced input from others. These findings demonstrate how expanding participation and fostering open communication in a hierarchical setting can lead to a shift in power dynamics—from one characterised by hierarchy and dominance to one of shared responsibility and collective purpose (see Box 4).

### PT 5: Role clarity and conflict resolution

**Box 5. Refined PT 5**

In hierarchical health system structures where roles and responsibilities are not clearly defined within organisational policies or guidelines, confusion and conflict often arise among sectors, implementation teams and individuals. This lack of clarity enables more resourceful or larger sectors to exert power over less influential ones, leading to frustration and uncertainty among participants from weaker sectors. As a result, work inefficiency, diminished accountability, and a sense of being overwhelmed emerge, particularly for those in vulnerable positions. The ambiguity in roles also creates opportunities for power imbalances, where individuals or sectors with perceived authority arbitrarily assign tasks beyond the original scope of responsibilities, further exacerbating conflicts and creating resentment. This dynamic undermines both efficiency and effective collaboration, with those in weaker positions bearing a disproportionate burden.

This PT posits that the absence of well-defined roles and responsibilities within organisational policies and guidelines can lead to conflicts, confusion, and inefficiency among individuals, teams, and sectors. This lack of clarity can result in poor engagement, dominance by more powerful sectors, and reduced accountability, ultimately hindering the implementation of programmes. Participants’ insights, supported by six CMOCs, highlighted how power imbalances and undefined roles exacerbate these challenges. For instance, Participant 9 described the consequences of an undefined job scope:

*“Yes, there is no limit or boundary to my job. I become like a multipurpose worker in this office; there is too much of a workload. I cannot say no to extra work since I am on a contractual post” (Participant 9)*

This statement from Participant 9 captures the overwhelming workload and inefficiency that arise from an undefined job scope. Without clear role definitions, individuals are often tasked with a wide variety of responsibilities, many of which fall outside their primary job duties. This situation leads to them being seen as ’multipurpose workers,’ attracting other sectors to offload additional tasks onto them, which dilutes their capacity to focus on core responsibilities. Observations during the study indicated that this issue was particularly pronounced at the block level, where teams are smaller, and many block coordinators took on tasks beyond their designated roles. A key factor contributing to this dynamic is the contractual employment status of many staff members.

Contractual employees often lack the job security that permanent staff enjoy, making them more vulnerable to exploitation. Without the authority to push back against additional tasks imposed by other sectors, these staff members feel pressured to accept extra work to maintain job security and avoid potential repercussions, such as non-renewal of their contracts. The absence of job security in these positions fosters an environment where contractual employees are more likely to comply with requests, even when the tasks fall outside their scope of expertise or responsibility. This dynamic not only exacerbates power imbalances but also leads to inefficiency and burnout, as these individuals become overburdened, carrying out multiple responsibilities without sufficient support or authority to contest these arbitrary assignments. Contractual employees often perceive that refusing such tasks could negatively affect their reputation or future employment prospects, further limiting their ability to negotiate workload boundaries.

The consequences of unclear roles were further illustrated by Participant 6, who initially faced confusion due to an undefined role.

*“Initially, there was a lack of clarity regarding my role leading to some confusion. I primarily focused on data management and reporting.Over time, extra tasks kept coming, and keeping convergence alive became my main job. It made me realise how much this depends on steady coordination and genuine collaboration with many actors. my.” (Participant 6)*

Participant 6’s account reflects the initial confusion that can arise when roles are not clearly defined. Over time, the participant adapted by taking on additional coordination responsibilities, which were not part of the original job scope. It became apparent from participant accounts and field observations that without clear role definitions, individuals must rely on ad-hoc coordination and personal initiative to navigate their responsibilities. This reliance on informal solutions often leads to the dominance of those who can better negotiate their roles, leaving others with a disproportionate burden. Participant 5 emphasised the need for predefined roles and responsibilities to facilitate smoother collaboration across sectors:

*“We need better-defined responsibilities from the start. If everyone knows their part, there will be less confusion, and there will be no unnecessary extra work, and it will be easier to work together.” (Participant 5)*

This suggestion from Participant 5 highlights the importance of establishing explicit roles and responsibilities within organisational policies to prevent confusion and conflict. Field observations during the study reinforced this insight, revealing that clear role definitions reduce misunderstandings and enable more efficient coordination. Without clear definitions, power struggles arise. Dominant sectors or individuals push extra tasks onto colleagues with less authority. This deepens workload imbalances. The emphasis on role clarity serves as a mechanism to balance power dynamics within ISC.

By defining roles explicitly, the potential for dominance by any one sector or individual is reduced, and responsibilities are more evenly distributed. Clear role definitions also mitigate the risk of one sector holding unchecked authority or bearing an undue burden, fostering a more equitable and efficient collaboration. These insights, derived from interviews, observations, and reflective analysis, have refined the understanding of how role clarity and hierarchical structures interact within ISC. Therefore, this programme theory was refined to reflect that ensuring role clarity from the outset is the critical mechanism for addressing these challenges, reducing power imbalances and fostering more effective and equitable collaboration across sectors (see Box 5).

### PT 6: Interpersonal connectedness

**Box 6. Refined PT 6**

In settings where participants establish personal connections beyond their formal professional roles, an environment of mutual trust and respect is fostered. This reduces hierarchical barriers, allowing participants to view their colleagues as partners rather than representatives of distinct sectors. The trust developed through these interpersonal relationships encourages participants to share resources more freely, provide mutual support, and engage in more open, candid communication. These dynamics promote a sense of camaraderie and shared purpose, enabling participants to navigate beyond rigid formalities and act with greater flexibility and spontaneity. As a result, teamwork improves, collaboration becomes more fluid, and intersectoral health interventions are implemented more effectively.

This PT posits that personal connections beyond professional boundaries reduce power imbalances by building trust and mutual respect, thereby enhancing ISC. Participants provided insights that generally support this theory, demonstrating that informal relationships foster a willingness to share resources and support one another, which helps balance power dynamics and leads to better teamwork and programme success. This theory was supported by 11 CMOCs derived from both interviews and field observations conducted during the study. Participant 14 highlighted the significance of personal relationships in fostering effective collaboration:

*“I know (named) officer; he really works hard to bring us all together. His intentions are good, but he is in this complex system, so he also has limitations. His house is nearer to mine, and we do meet often over tea and discuss a lot many things which we don’t discuss in meetings, since we know each other well it’s easier to work together more closely.” (Participant 14)*

This account illustrates how interpersonal relationships can soften the rigid effects of formal hierarchical boundaries. Both participant accounts and observations (particularly during after-office coffee and tea meetups) highlighted that meetings outside the constraints of professional settings provide opportunities for more candid and genuine discussions. In these informal spaces, the rigidity of formal protocols is lessened, enabling individuals to communicate more openly. This dynamic helps reduce power imbalances by creating a context where authority is less pronounced and participants feel more comfortable sharing their thoughts.

Participant 14’s frequent informal interactions foster mutual respect and understanding, facilitating a more inclusive and collaborative environment that is often difficult to achieve in formal meetings. By fostering these personal connections, the hierarchical barriers that often restrict open communication are softened, leading to more balanced decision-making and better collaborative action in ISC. Similarly, Participant 13 highlighted how personal relationships impact programme implementation:

*“For example, recently I facilitated a session on managing underweight children at a community event without formal invitation just based on the personal request. This spontaneous action was well-received, showcasing the flexibility and mutual support among colleagues.” (Participant 13)*

Participant 13’s narrative illustrates how personal relationships promote spontaneity and adaptability, which are crucial for overcoming the limitations imposed by formal procedures. In the above illustrative example, participants did not rely on formal invitations but instead utilised informal connections to organise and conduct the training. This proactive approach reflects how personal connections enable swift and effective responses, bypassing bureaucratic constraints that could otherwise delay programme implementation.

The mutual support among colleagues, fostered by these personal relationships, helped create a more egalitarian environment where hierarchical power dynamics were less pronounced. This environment enabled a more cohesive and effective ISC, as all stakeholders felt empowered to contribute to collective goals. The interviews and observations point to generative causation. Within formal hierarchies, relationships reduce rigidity, foster trust, and strengthen communication. This lowers power effects and supports better collaboration (see Box 6).

### Theory refinement based on the participants’ views

Integrating case study findings through CMO configuration into the refinement of Programme Theories (PTs) has provided a robust framework for understanding the dynamics of ISC. An example of theory refinement mentioned in Table 3. Dissenting perspectives were present across several interviews. Some participants described inclusive and empowering processes, while others viewed meetings as symbolic or procedural. These contrasting views were treated as disconfirming evidence rather than anomalies. Each contradiction was used to refine mechanisms and define boundary conditions. For instance, when collaboration was perceived as genuine in one block but tokenistic in another, the analysis identified contextual factors, such as leadership consistency or resource control, that explained these differences. Recognising both supportive and dissenting views helped strengthen the critical interpretation and ensure that the refined theories reflected the diversity of real-world experiences. Observational data played a critical role in validating these refinements, adding a layer of contextual and practical nuance to participant insights.

For PT 1, initially, the theory emphasised equal ownership. However, practical insights from participants, supported by observations of how certain sectors dominated meetings, demonstrated the importance of equitable ownership instead of equal ownership. Recognising pre-existing inequalities and ensuring all sectors have a voice are crucial for genuine collaboration. Committees and task forces observed during the implementation showed that equitable ownership enhances participation, coordination, and meaningful engagement beyond procedural formalities.

In PT 2, the refinement incorporated the nuances of proactive and consistent leadership. Initially, the focus was on democratic leadership, but participant insights, corroborated by observations of leadership behaviours during meetings, highlighted the critical importance of follow-up, proactive engagement, and responsiveness. Observational data provided additional context on how leadership engagement, or lack thereof, influenced the overall collaboration dynamic. The refined theory emphasises sustained, stable leadership to protect a collaborative culture through transitions. This fosters trust and keeps power in check.

For PT 3, the refinement focused on equitable resource allocation, drawing from participants’ experiences with resource sharing. Originally centred on fair resource distribution to mitigate power imbalances, the refined theory now also recognises and leverages unique sector strengths for collaboration. Examples of this, such as the use of untied funds for women’s empowerment and addressing nutritional deficiencies, illustrated how equitable resource allocation fostered sustainability and enhanced sectoral engagement.

In PT 4, which focuses on hierarchical structures, the refinement addressed the need to mitigate issues of disrespect and distrust that hinder communication. Observations of power dynamics during meetings, where hierarchical dominance affected participation, informed the refinement. The refined theory now includes strategies like predetermined discussion topics and transparent performance indicators to reduce hierarchical dominance, foster open communication, and prioritise collective decision-making, ultimately enhancing engagement.

PT 5 was refined to focus on role clarity as a dynamic and ongoing process rather than a static one. Participants’ experiences with role ambiguity and job scope challenges, observed in the day-to-day functioning of block-level teams, highlighted the negative impacts on efficiency and engagement. he refined theory now emphasises continuous coordination and proactive problem-solving to mitigate conflicts, enhance accountability, and reduce power struggles, fostering better collaboration.

Finally, PT 6, which initially highlighted the positive impact of personal connections on trust and resource sharing, was refined to balance the benefits of informal relationships with maintaining professional boundaries. Participants’ experiences with spontaneous, informal gatherings, which often lead to practical actions,demonstrated the potential benefits of these relationships. However, the need to mitigate potential conflicts with established hierarchies was also evident in the observations. The refinement ensures a comprehensive understanding of the complexities of interpersonal relationships, promoting effective collaboration while managing professional boundaries. Incorporating observational data into the refinement of these PTs added an important dimension, ensuring that the PTs not only reflect participants’ views but are also grounded in the real-world behaviours and dynamics observed throughout the study.

## Discussion

This case study’s findings explore the pivotal role in shaping ISC within the NNM of Assam, India. The study unpacks how, why, for whom, in what context, and to what extent power impacts ISC. Recognising and addressing power differentials among different sectors is essential for successful ISC, as it ensures equitable participation and decision-making. This involves actively engaging participants in shaping collaborative efforts. The PTs, refined through interviews with participants and field observations, offer insights to enhance collaborative governance and partnership effectiveness across sectors that are transferable to other settings.

ISC in health systems is characterised by multifaceted interactions among various participants, sectors, and levels of governance. The findings from this case study propose six refined programme theories, demonstrating how diverse factors interconnect to shape effective collaboration. The refined PT on equitable ownership aligns with the broader literature on inclusive policy development, which highlight the importance of extensive engagement across sectors to enhance mutual understanding and coordination [105–110]. The formation of task forces and committees by the government machinery, rather than acting as barriers, served as a mechanism for institutional power to bring diverse sectors together. Over time, through repeated meetings and interactions, this process naturally fostered open communication, trust, and interpersonal relationships. These interactions created informal spaces for dialogue, ultimately cultivating a sense of collaboration and mutual understanding among stakeholders. However, the study also reveals challenges in achieving genuine collaboration, as some meetings serve merely as formalities.

This echoes concerns in the literature about the gap between theoretical frameworks and practical implementation, where formal structures exist but fail to produce substantive collaboration [111–113]. This suggests a need for more participatory meeting structures to ensure meaningful engagement. The emphasis on equitable rather than equal ownership marks a significant shift in collaborative dynamics. The distinction is echoed by others who argue that equity-focused approaches are more effective in addressing power imbalances [114,115]. This perspective is essential for enhancing participation and fostering shared responsibility. Our findings align with Arnstein’s [116], broader theory of genuine participation, which advocates for true power-sharing rather than tokenistic involvement. The procedural formalities observed in the case study contrast with this ideal, further highlighting the need for flexible, participatory approaches that prioritise engagement over mere compliance.

Leadership support and goal alignment are vital factors in fostering collaboration. According to Transformational Leadership Theory [117], leaders who inspire and motivate staff towards a shared vision are critical for sustaining collaborative efforts. The study’s findings, which show how leadership can influence participation and trust, resonate with the broader literature, emphasising that leadership is a key determinant of successful collaboration [118,119]. Yet, leadership alone cannot overcome systemic barriers to collaboration. Other factors, such as bureaucratic processes and local-level engagement issues, can still hinder progress, even with strong leadership in place. The PT on equitable resource allocation resonates with Rawls’ Theory of Justice, advocating for fairness in resource distribution [120].

Rawls’ principles are mirrored in participants’ calls for recognising sector strengths and promoting resource sharing. Practical examples, such as using untied funds for broader community needs, reflect how equitable resource allocation can mitigate power imbalances and enhance collaboration. Balancing resources across sectors ensures that no single sector dominates, aligning with broader studies on effective resource distribution [102,104].

The challenges related to communication and trust in hierarchical structures are consistent with Schein’s Organisational Culture Theory [121], which stresses the role of organisational culture in shaping communication and trust. Our study’s emphasis on fostering inclusive dialogue and collective decision-making addresses the need for transparent and respectful communication. In contrast, the observed bureaucratic processes and lack of genuine engagement echo findings from studies that highlight how rigid organisational cultures can hinder collaboration, despite efforts to foster trust. Lastly, role clarity emerged as a key factor influencing collaboration. The study’s findings align with Hackman and Oldham’s Job Characteristics Model which posits that clearly defined roles improve job satisfaction and organisational effectiveness [122]. Participants’ experiences of role ambiguity illustrate how unclear roles can exacerbate power imbalances and lead to inefficiency. In contrast, fluid roles, as seen in some collaborative environments, can foster innovation and problem-solving [123]. This balance between role clarity and flexibility is crucial for managing power dynamics within teams.

### Theoretical and practical implications

This study significantly advances the theoretical understanding of the complex interplay between power dynamics, context, and outcomes. The refined programme theories developed from empirical data highlight the importance of equitable participation, leadership support, resource allocation, communication and trust, role clarity, and interpersonal connections. These PTs provide a robust analytical tool for examining ISC across diverse settings, addressing gaps in the literature regarding the operationalisation of power within ISC.

This study offers practical and actionable insights for policymakers and practitioners. The findings emphasise the need for inclusive policy development, integrating diverse sectoral perspectives to enhance coordination and stakeholder ownership. Leadership plays a pivotal role in sustaining collaborative cultures through motivation, empowerment, and goal alignment. Equitable resource allocation is crucial for mitigating power imbalances and fostering engagement. Effective communication and trust-building practices are essential for overcoming hierarchical barriers and ensuring stakeholder participation. The contributions of this study have significant implications for future research. The refined programme theories provide a framework for comparative studies and cross-regional analyses, allowing researchers to further explore how power dynamics shape collaborative outcomes.

### Observational insights on power dynamics and ISC

Observational data, including non-verbal cues and informal exchanges, provided critical insights into stakeholder relationships and power dynamics, enriching the findings from in-depth interviews. Observations revealed that power imbalances, particularly in hierarchically dominant sectors like health and public administration, often limited meaningful participation from less powerful sectors, such as social services. For instance, block-level officials were frequently interrupted by higher authorities requesting updates, highlighting how power structures constrained discussions and reinforced sectoral silos. Observational data further validated interview findings, particularly around bureaucratic delays and power asymmetries, such as delays in approvals from dominant sectors that hindered cross-sectoral progress. Informal interactions among stakeholders in some blocks demonstrated collaboration beyond formal mechanisms, illustrating how stakeholders navigated formal barriers to foster intersectoral collaboration. This observational data, triangulated with interview findings, helped refine Programme Theories by revealing how contextual factors like leadership, resource distribution, and trust levels influenced collaboration dynamics. These insights highlight the importance of addressing power imbalances and enhancing trust-building measures to strengthen intersectoral collaboration.

## Conclusion

These findings highlight the need to address structural and relational mechanisms to improve ISC outcomes. By highlighting the importance of a balanced approach that integrates these elements, the study offers research and practical insights for advancing the implementation of intersectoral health policies and collaborative health interventions. Key themes such as inclusive policy development, leadership support, equitable resource allocation, communication, trust, and role clarity were essential in fostering sustainable and effective ISC. The findings emphasise the significance of equitable participation and decision-making to enhance mutual understanding and coordination among stakeholders, while leadership and resource allocation are crucial for addressing power imbalances and ensuring continuous support. Additionally, transparent communication, trust-building, and well-defined roles were vital for improving collaboration and organisational efficiency. The study’s insights provide a practical roadmap for fostering successful ISC by leveraging both structural and relational factors. Looking ahead, researchers could build on the refined PTs proposed in this study to develop a middle-range theory that explains the influence of power in ISC within health system research. Such a theory would offer a deeper understanding of how these dynamics influence collaboration and provide a framework for addressing challenges in ISC implementation.

### Limitations of the study

The evaluation primarily focused on qualitative interviews, and the absence of quantitative data or mixed methods could be seen as a limitation in capturing a fuller picture of the ISC process. While realist evaluations often incorporate mixed methods to enrich findings, this study intentionally focused on in-depth interviews that were guided by PTs, which might have missed insights from non-participant groups or other forms of data. The findings come from one district case, predominantly from programme implementors, in Assam and reflect that specific administrative and social context. The refined programme theories are context-specific explanations, not universal models. Their use lies in helping others think about similar settings rather than applying them directly elsewhere. Future research could test and refine these theories in other districts or states, using mixed methods to strengthen and compare the results.

## Supporting information

S1 Text Candidate Initial Programme Theories. (DOCX)

S2 Text List of rest 62 CMOCS. (DOCX)

S3 Text PT refinement process for PT 2 to PT 6. (DOCX)

S1 Checklist Inclusivity in global research. (PDF)
