# Supplementary material for: Understanding the influence of power dynamics in intersectoral collaboration: A realist evaluation in Assam, India
Source: PLOS Glob Public Health. 2025 Dec 12;5(12):e0005639. doi: 10.1371/journal.pgph.0005639 (PMC12700422; doi:10.1371/journal.pgph.0005639)
Supplement: S1 Text — (DOCX) [file pgph.0005639.s001.docx]

**Supplementary file: 1 Candidate Initial Programme Theories**

| IPT 1:  Fair opportunity & Participation | During the policy designing process, IF different sectors/departments are given equal opportunity  THEN sectors feel valued, acknowledged and empowered LEADING TO increases the participation, coordination, and collaboration among stakeholders of different sectors. |
| --- | --- |
| IPT 2  Leadership support | IF in settings where leadership structures are non-hierarchical and sector goals are aligned with the sectoral priorities THEN this motivates and engages staff, creating a sense of team efficacy and a shared sense of responsibility. LEADING TO Enhanced connectedness with the broader system and is more likely to result in better engagement/collaborative action |
| IPT 3  Fair Resource | IF the resources are fairly distributed and made accessible to all the sectors, THEN it decreases the dependency of weaker sectors and balances out the perceived power imbalances LEADING TO improved engagement and collaborated action. |
| IPT 4  Platforms | In settings where hierarchical structures exist, and during collaborative meetings platforms IF stakeholders are given fair chance to express their views THEN stakeholders feel valued, motivates and brings in a sense of shared accountability LEADING TO A greater degree of participation, coordination and collaborated action |
| IPT 5 Role delineation | IF In settings where role clarity and tasks are sufficiently delineated in policy guidelines, THEN it reduces conflict and/or confusion among sectors and implementing teams, LEADING TO lead efficient task completion and greater accountability that will positively impact the collaborative engagements |
