# Supplementary material for: Understanding the influence of power dynamics in intersectoral collaboration: A realist evaluation in Assam, India
Source: PLOS Glob Public Health. 2025 Dec 12;5(12):e0005639. doi: 10.1371/journal.pgph.0005639 (PMC12700422; doi:10.1371/journal.pgph.0005639)
Supplement: S2 Text — (DOCX) [file pgph.0005639.s002.docx]

**Supplementary file 2:** List of rest 62 CMOCS

| **CMOC No** | **Context** | **Mechanism** | | **Outcome** | **Source** |
| --- | --- | --- | --- | --- | --- |
|  |  | **Resource** | **Reasoning** |  |  |
| CMOC 1 | Health departments dominate discussions in intersectoral meetings due to their perceived authority. | Meeting platforms, leadership from health officials, and budgetary control. | The dominance of the health sector discourages other departments from voicing their opinions. | Reduced participation from less powerful sectors, leading to imbalanced collaboration. | *"Whenever we have a meeting, it always feels like the health department has the final word. No matter what we from WASH or ICDS say, it’s like our ideas don’t really count. It’s frustrating, you know? After a while, it makes us feel like there’s no point in speaking up because no one’s really listening. It’s hard to stay motivated when it feels like only one side matters in the end." -Participant 3  "You know, the meetings we attend are meant to be spaces where everyone contributes, but the reality is quite different. Our sector, ICDS, works so closely with the community, especially mothers and children, so we bring a lot of ground-level insights. But when we try to share those in the meetings, it feels like they’re overlooked. The health department always seems to dominate the conversation, steering it towards their priorities. -Participant 9* |
| CMOC 2 | Limited resource allocation for certain sectors, such as social welfare, impacts their participation. | Unequal funding distribution and lack of decision-making authority for smaller sectors. | Resource-poor sectors feel dependent on well-funded ones, exacerbating power imbalances. | Poor collaboration and strained relationships among sectors. | *"Look, we’re always the ones left waiting, and it’s exhausting. They keep saying, ‘Oh, we’ll include you in the next plan,’ but the reality is, we don’t even get a proper budget to run our programmes. How can we participate when we don’t have the basics? Then they come to us saying, ‘Social welfare isn’t performing well.’ Well, how do you expect us to perform without funds? It’s like asking someone to cook a feast but not giving them ingredients! - Participant 6  "You know what the problem is? We depend on the health department for everything—funds, approvals, you name it. And they act like they’re doing us a huge favour. But when it’s time to make decisions about nutrition programmes, they don’t even look our way! – Participant 14  I remember one meeting where we suggested adding more funds to tackle severe malnutrition. You know what they said? ‘We’ll see if there’s leftover money.’ Leftover money? Are we running a programme or begging for scraps? It’s insulting, and it just creates tension. How can we work together when we’re always made to feel like we’re begging for something that should already be ours?"- Participant 12* |
| CMOC 3 | Collaborative planning sessions where each sector has equal opportunity to highlight resource needs. | Structured forums with time allocated for all sectors to share priorities and gaps. | Inclusive discussions create a sense of ownership and respect, reducing tensions related to resource imbalances. | Strengthened collaboration and more equitable resource allocation decisions. | *"You know, earlier, planning meetings used to feel like a one-sided affair. Health would present its requirements first, and before anyone else could properly put their points across, the budget would already be allocated. Other sectors like WASH and ICDS barely got a chance to make their case. It wasn’t intentional, but it was happening, and it created tension. – Participant 11*  *Now, things are different. The leadership ensures that every department gets equal time to talk about what they need. Last time, WASH spoke about how sanitation issues were affecting nutrition outcomes, and instead of brushing it off, we actually had a serious discussion about re-allocating some funds to fix it. It felt like real teamwork for the first time. When everyone gets a say, the decisions are stronger, and the results are better."- Participant 13  "Earlier, planning meetings were full of unspoken tensions. The departments with more power, more funding, and more say would dominate, and the others would just go along with it. It wasn’t fair, and it definitely wasn’t effective. But the way we do things now has changed the game. Participant 1*  *Now, everyone gets structured time to speak. It sounds like a small thing, but it makes a huge difference. When we went sector by sector in the last meeting, we actually identified a major gap—funds were being over-allocated to outreach activities while school-based interventions were underfunded. Because we had open discussions, we were able to shift things around to balance it better. That’s the power of fair discussions—everyone gets what they need, not just what the powerful sectors decide." Participant 1* |
| CMOC 4 | Convergence meetings are regular but discussions risk becoming repetitive. | Government **prioritisation of collaboration** ensures frequent meetings to address collective goals. | Stakeholders feel **engaged but seek variety** in the topics discussed to remain fully committed to the process. | Reduced **repetition in discussions** and enhanced relevance of collaborative efforts. | ***"Initially, meetings felt repetitive, but as convergence meetings became regular, all sectors started seeing the value of collaborative efforts. Discussions are now more aligned with action plans that include diverse perspectives." (Participant 13)*** *At first, these convergence meetings felt like we were just going over the same points again and again, and it was hard to stay engaged. But as they became more structured, things started changing. In our last meeting, we discussed school health interventions, and for the first time, it felt like every sector brought something meaningful to the table. The discussions were focused on action—like aligning health check-ups with mid-day meal programmes—and it finally feels like we’re making real progress together." – Participant 8* ***(*Key words*: repetitive, value, aligned, perspectives).*** |
| CMOC 5 | Frequent leadership changes at the block level disrupt continuity in intersectoral collaboration. | Inconsistent leadership priorities, lack of institutional memory, and limited knowledge transfer. | Stakeholders feel uncertain about the direction of programmes and frustrated by the lack of stable leadership. | Collaboration efforts become fragmented, reducing programme effectiveness. | *"You know, just when we start getting things in order, a new officer comes in, and boom—everything changes. It’s like we’re always running in circles. The last officer had finally understood how crucial our role was in addressing malnutrition. We had started some good joint initiatives with health and WASH. But then a new person took charge, and suddenly, their focus was something else.*  *Now, we have to sit through more meetings explaining the same things all over again. What’s worse is that some programmes that were working well just get abandoned because the new leadership wants to ‘restructure’ everything. It’s frustrating. By the time we all adjust, another officer will probably come in, and we’ll be back to square one."*  *– Participant 14  "I’ve lost count of how many times we’ve had to reintroduce ourselves and our work to a new leader. Every time someone new takes charge, they come in with fresh enthusiasm, but they also want to ‘revamp’ things without really understanding what’s already working. It’s not their fault—they’re just trying to leave their mark—but it slows everything down.*  *For example, we had finally established a strong coordination mechanism between health, ICDS, and social welfare. Meetings were happening regularly, and we were seeing real progress. Then a new officer came in and decided to reshuffle priorities. Suddenly, we had to put everything on hold and go through weeks of ‘review’ meetings. By the time they understood what was needed, we had lost months of progress. It’s exhausting."*  *– Participant 7* |
| CMOC 6 | Community representatives are included in meetings but are rarely given opportunities to lead discussions. | Platforms for participation, but no provision for equitable power-sharing mechanisms. | Community members feel undervalued, perceiving their role as tokenistic rather than meaningful. | Weak engagement of community representatives and missed opportunities for localised insights. | *""They invite us to meetings, make us sit there for hours, but when it’s time to talk, they’ve already made all the decisions. Sometimes, they’ll ask, ‘Does anyone have anything to add?’ but by then, everything is already finalised. What’s the point of speaking when no one actually listens?*  *Last month, I tried to bring up an issue about how some anganwadi centres weren’t getting enough food supplies. But before I could even finish, someone from health jumped in with another topic, and that was it. No one asked me to continue. It’s frustrating because we are the ones who see what’s happening on the ground every day. If they don’t hear us out, how will things ever improve?"*  *– Participant 10* |
| CMOC 7 | Frequent meetings led by senior officials but designed to include inputs from all sectors. | Structured **agendas and follow-ups** ensure clear roles and responsibilities for all stakeholders. | Stakeholders experience **role clarity** and feel their contributions are essential to the programme's success. | Enhanced **coordination and shared ownership**, fostering a balanced environment. | ***"Frequent meetings with clearly defined roles and follow-ups have helped clarify our responsibilities and fostered mutual respect among departments. This makes collaboration more meaningful."*** *(Participant 11)*  *"These committees have given us a space to talk about the real problems in our villages. Before, it always felt like decisions were made far away, without any input from the community. But now, we’re invited to these meetings, and they ask for our perspectives. Last time, I raised concerns about malnourished children not getting regular Anganwadi rations, and the issue was taken seriously. It’s empowering to know that what we say actually matters." (Participant 4)*  ***(*Key words*: roles, responsibilities, mutual respect, meaningful).*** |
| CMOC 8 | Leadership facilitates joint action plans across health, ICDS, and WASH sectors to tackle malnutrition. | Joint planning workshops, regular updates, and collaborative frameworks shared across sectors. | Inclusive leadership fosters trust and mutual respect, encouraging sectors to work together effectively. | Improved coordination and alignment of sectoral goals, leading to better programme outcomes. | *"I remember when we used to work in silos—health was doing its thing, ICDS had its own plans, and WASH was just expected to ‘support’ when needed. There was no real coordination, and honestly, it led to a lot of wasted effort. But ever since the leadership started holding these joint planning workshops, things have changed. Now, we actually sit together, discuss our challenges, and make proper plans where each sector has a clear role.*  *Last month, we identified a village with high malnutrition rates. Instead of just pushing health interventions, we brought in ICDS to strengthen supplementary nutrition, and WASH ensured the drinking water supply was safe. This approach is working much better because we are fixing the problem from all sides. It’s a relief to finally have a system where we aren’t stepping on each other’s toes but actually moving forward together."*  *– Participant 3,   "In the past, when we heard about government ‘plans,’ they felt distant—something decided in offices, not in the villages where the problems actually exist. But now, with these joint planning meetings, even community members like us get to sit at the table. It makes a big difference because we see things on the ground that officials might miss.*  *For example, in our area, children were getting sick even after receiving nutritional support. When we raised this in the meeting, ANMs helped. If we hadn’t been there, this issue might have been ignored, and they would have only focused on something else. Leadership encouraging this kind of teamwork is what makes real change happen."*  *– Participant 12,* |
| CMOC 9 | Health departments dominate resource allocation decisions for community-level nutrition programmes. | Larger budgets and decision-making authority concentrated within the health sector. | Stakeholders from other sectors feel sidelined, leading to frustrations and disengagement from collaborations. | Power asymmetry weakens trust and reduces the effectiveness of intersectoral programmes. | *"I don’t understand why the health department gets to call all the shots when it comes to nutrition programmes. We in social welfare work directly with the most vulnerable families—pregnant women, children, and undernourished individuals. We see the challenges up close, but when it comes to budget discussions, we are barely consulted.*  *Last year, we requested funds for a special nutrition outreach programme for adolescent girls, but the health officials decided to prioritise medical check-ups instead. Of course, check-ups are important, but how does it make sense to diagnose malnutrition without providing enough support to fix it? It’s frustrating because we are supposed to be working together, but in reality, we’re just watching from the sidelines."*  *– Participant 6,* |
| CMOC 10 | Convergence meetings align multiple sectors to jointly plan and execute health initiatives. | Government provides **inclusive platforms** for collaboration, integrating inputs from various stakeholders. | Stakeholders perceive **genuine engagement** and appreciate that their perspectives are valued equally. | Improved **coordination, reduced power imbalances**, and meaningful intersectoral collaboration. | ***"Regular convergence meetings have improved participation from all stakeholders, and the inclusion of every department’s input has created a sense of shared ownership." (Participant 5)   (*Key words*: participation, input, shared ownership).*** |
| CMOC 11 | Informal communication channels like WhatsApp groups complement hierarchical decision-making structures. | Access to instant messaging platforms allows faster resolution of issues and informal updates. | Informal channels reduce delays caused by bureaucracy and make collaboration more efficient. | Enhanced coordination and trust among stakeholders, enabling quicker decision-making. | *"You won’t believe how many issues get sorted out on WhatsApp before they even make it to a formal meeting. Earlier, if we needed a small clarification or approval, we had to send an official letter, wait for signatures, and by the time the response came, the problem had either grown bigger or become irrelevant. But now, if there's an urgent issue—like a delay in ration distribution or a health camp needing quick approvals—one message on WhatsApp, and we get an answer in minutes.*  *The best part is that it keeps everyone in the loop. Instead of waiting for the next meeting to bring up a problem, we just flag it immediately, and someone from another sector might even offer a quick fix. It has made collaboration much faster and more efficient. Of course, we still need formal processes, but WhatsApp makes sure work doesn’t stop because of paperwork."*  *– Participant 14,   "Honestly, WhatsApp has made my job so much easier. Before, we’d be stuck waiting for formal emails or letters to get responses, and sometimes even getting a meeting scheduled would take weeks. But now, if I need an update on a programme, I just drop a message in the group, and within hours—sometimes minutes—I have an answer.*  *It’s also helped build relationships. When you’re only interacting with people in meetings, everything feels formal and rigid. But when you’re exchanging quick updates on WhatsApp, there’s a sense of teamwork. It feels like we’re all working towards the same goal instead of just ticking off bureaucratic requirements. And let’s be honest—half the time, decisions that take weeks through official channels get sorted out in a single WhatsApp chat!"*  *– Participant 16* |
| CMOC 12 | Emphasis on collaboration through structured committees and task forces in a hierarchical system. | Regular meetings and task forces provide **formal channels** for participation and decision-making. | Stakeholders feel **empowered and valued** as their input is actively sought and incorporated into discussions. | Increased **trust, empowerment, and collaboration** across sectors, reducing dependency on dominant actors. | *"Over the last couple of years, the government has placed significant emphasis on collaboration, forming committees and task forces. These committees have given us a platform to share our ideas and feel more involved in decisions." (Participant 13)* "*When these committees were first formed, I wasn’t sure if they would actually work. But now, with regular meetings, we’re finally seeing some collaboration. For example, in the last meeting, social services got to highlight the gaps in welfare schemes that were affecting nutrition outcomes. Our suggestions were not only heard but actually included in the final action plan. It feels good to know that our sector has a voice and that we’re contributing meaningfully to the process."* ***(Participant 4)*** *(****Key words:*** *emphasis on collaboration, platform, involved).* |
| CMOC 13 | Convergence meetings focus on specific, actionable topics to maintain engagement across sectors. | Well-structured agendas, action points, and frequent follow-ups by leadership. | Focused discussions ensure stakeholders stay engaged and see the relevance of their contributions. | Increased stakeholder participation and more actionable outcomes from collaborative efforts. | *"I remember how frustrating these meetings used to be—endless discussions, everyone talking in circles, and in the end, no real decisions. It felt like we were attending meetings just for the sake of it. But things have changed now. The leadership makes sure there’s a clear agenda, and every sector knows what they need to contribute. We come in, discuss what actually matters, and leave with action points instead of just ideas floating in the air. Last month, we had a convergence meeting on school nutrition, and instead of just hearing updates, we broke into small groups, set priorities, and assigned responsibilities on the spot. Within a week, I saw follow-ups happening—schools getting water filters repaired, ICDS linking mid-day meals with health check-ups. When meetings are structured well, people actually feel like their time is valued, and things get done."*  *– Participant 9*  *"Before, these meetings felt like a formality—just long discussions where nothing really changed on the ground. But now, with proper agendas and clear follow-ups, we actually see results. Last time, when we raised the issue of children missing health check-ups due to school timings, instead of just noting it down, the officials immediately worked out a plan with the health and education teams to schedule camps on weekends. That’s what we need—meetings where problems are actually solved, not just discussed."* |
| CMOC 14 | Equitable distribution of untied funds across sectors at the block level. | Untied funds allocated proportionately to all sectors, enabling each to address its specific needs | Stakeholders feel their needs are recognised, fostering trust and fairness in collaboration. | Increased autonomy of less-resourced sectors, leading to improved collaboration and reduced power imbalances. | *"For the longest time, we had to rely on whatever was left after the bigger departments took their share, and honestly, it felt like we were just tagging along. But now, with untied funds being distributed fairly, we finally have the freedom to plan according to our actual needs. Last month, we used part of our allocation to provide emergency nutritional support to vulnerable families without waiting for approvals from other sectors. It’s a big shift. Public administration also benefits because it no longer has to keep mediating between sectors fighting over resources. When funding is fair, collaboration becomes smoother—no more back-and-forth delays or departments feeling sidelined."* |
| CMOC 15 | Public representatives feel constrained by the lack of authority in the existing framework of intersectoral meetings. | The hierarchical structure limits **decision-making power** of less influential stakeholders. | Stakeholders feel their **contributions are tokenistic**, reducing their willingness to engage further. | Reduced **trust and openness**, hampering effective collaboration. | **"As a public representative member, I don’t have much authority within the existing framework, I can only push things up to some extent."** (Participant 16) |
| CMOC 16 | Meetings do not encourage open communication or mutual respect among sectors. | The dominance of powerful sectors discourages **two-way communication** and creates a one-sided dynamic. | Stakeholders feel **hesitant to express their views**, leading to a lack of diverse perspectives in decisions. | Poor **information sharing**, with narrow and sector-biased decision-making. | **"In meetings, it’s a cacophony of voices—everyone talks, but few truly listen... The health department’s influence looms large, shaping decisions in its favour while others struggle to break through the noise."** (Participant 14) |
| CMOC 17 | Leadership ensures that additional funds for nutrition initiatives are shared across all sectors. | Transparent processes for resource-sharing and equal access to financial allocations. | Clear and open distribution mechanisms reduce resentment and foster inclusivity among stakeholders. | Better participation from all sectors, reducing dependency and enhancing mutual respect in collaboration. | *"Before, it always felt like the health department controlled everything, and we just had to adjust with whatever little was left. But now, with leadership ensuring fair distribution, we actually get a say in how funds are used. This time, we were able to allocate resources specifically for social protection schemes that support malnourished children beyond just medical treatment. It finally feels like real collaboration, not just following orders." Participant 4   "In our village meetings, people used to ask why only health camps were getting support while other basic needs, like nutrition awareness and food security, were ignored. Now that funds are shared more equally, we can focus on long-term solutions, like setting up kitchen gardens for families. It’s good to see leadership making sure every sector gets what it needs instead of just one department taking charge of everything." Participant 12* |
| CMOC 18 | Joint procurement of essential supplies for nutrition and WASH programmes at the block level. | Shared procurement systems that meet the needs of all sectors, with equal representation in decision-making. | Collaborative resource management ensures smaller sectors are not overshadowed, promoting shared ownership. | Enhanced collaboration and trust, with a collective sense of accountability for resource use. | *"Earlier, procurement was a nightmare—each sector fighting for their own supplies, delays in approvals, and endless paperwork. Health would often get priority, while WASH and ICDS had to wait. But now, with joint procurement, everything is streamlined. We all sit together, list out what we need, and make collective decisions. It’s faster, more transparent, and no one feels sidelined. Plus, bulk purchasing has saved costs, which means more resources for actual implementation." – Participant 3  "In the past, WASH struggled to get enough sanitation kits for schools, while the health department had extra medical supplies that weren’t immediately used. Now that procurement is planned together, we can make sure every sector gets what’s needed without wastage. It’s good to see decisions being made fairly, with everyone involved. When resources are shared properly, it builds trust—we finally feel like equal partners, not just silent observers."* ***– Participant 13*** |
| CMOC 19 | Decentralised resource management ensures funds are allocated based on the specific needs of sectors. | Local-level input in budget decisions and resource allocation based on block-level priorities. | Decentralised decision-making empowers sectors to identify and address their own priorities without external bias. | Increased efficiency in resource use, improved collaboration, and reduced power hierarchies. | *"Before, most budget decisions were made at higher levels, and by the time the funds reached us, they were already earmarked for things that didn’t always match local needs. Now that decisions are happening at the block level, we finally have the flexibility to allocate resources where they’re actually needed. Last time, instead of blindly following a state-level directive, we adjusted funds to fix a critical shortage in nutrition supplies for remote villages. It’s made collaboration easier because each sector now has a real say in what gets prioritised." – Participant 15* |
| CMOC 20 | In settings with poorly defined roles, contractual staff feel overburdened with undefined or excessive tasks. | Lack of **role clarity** and **contractual constraints** forces staff to take on multipurpose responsibilities. | Staff feel **powerless to refuse additional tasks**, leading to exhaustion and reduced job satisfaction. | Reduced **efficiency** and poor **engagement** due to overwork and low morale. | **"Yes, there is no limit or boundary to my job. I become like a multipurpose worker in this office; there is too much of a workload. I cannot say no to extra work since I am on contractual post."** (Participant 9) |
| CMOC 21 | Staff are often assigned additional responsibilities outside their original scope of work. | Lack of **structured policies** or guidelines to define roles and responsibilities clearly. | Stakeholders experience **confusion** about their roles and tasks, making coordination and efficiency difficult. | Poor **coordination** and **reduced accountability**, leading to inefficiencies in programme implementation. | **"Initially, there was a lack of clarity regarding my role leading to some confusion. I primarily focused on data management and reporting. However, as time passed, I am dragged into so many other additional tasks."** (Participant 6) |
| CMOC 22 | Continuous coordination and collaboration are required to keep intersectoral processes functional | Over time, staff develop an **understanding of the importance of coordination** through lived experience. | Stakeholders feel **motivated to engage in coordination efforts**, despite the lack of clear guidelines. | Improved **interpersonal relationships** but at the cost of **efficiency** and **overwork**. | **"Major work was to keep the convergence alive. I slowly realised the importance of continuous coordination and collaboration with various stakeholders."** (Participant 6) |
| CMOC 23 | Equitable distribution of field staff across all programme areas. | Additional staff positions created to reduce workload disparities between sectors. | Balanced staffing reduces over-reliance on certain departments and ensures equal participation in interventions. | Improved programme implementation and more meaningful involvement of all sectors. | *"In the past, we used to see health staff everywhere, while other departments like ICDS and WASH barely had anyone on the ground. It felt like everything was falling on health, and the other sectors were just struggling to catch up. But now, with more staff hired across all departments, things are much more balanced. ICDS workers are able to handle their own responsibilities, like nutrition distribution, without constantly relying on health staff. It’s made the work smoother, and we’re seeing better coordination because everyone is pulling their weight."* ***– Participant 11*** |
| CMOC 24 | Collaborative planning sessions where each sector has equal opportunity to highlight resource needs. | Structured forums with time allocated for all sectors to share priorities and gaps. | Inclusive discussions create a sense of ownership and respect, reducing tensions related to resource imbalances. | Strengthened collaboration and more equitable resource allocation decisions. | *Earlier, planning meetings felt like a race, with the bigger sectors dominating the conversation and smaller ones like ours barely getting a chance to speak. Now, the leadership ensures every sector has equal time to present their challenges. Last month, we highlighted gaps in social protection schemes for malnourished families, and instead of being overlooked, it was addressed right there in the meeting. It feels fair now—decisions are based on actual needs, not just who has more power."* ***– Participant 7*** |
| CMOC 25 | Transparent reporting of resource usage by all sectors, monitored by a neutral third party. | Regular reporting frameworks and independent monitoring teams to review fund utilisation. | Transparency builds trust, ensuring all sectors feel accountable and valued in the collaboration process. | Improved trust and reduced conflicts, fostering a more egalitarian environment for intersectoral work. | *"In the past, there was always suspicion about how resources were being used—whether one department was taking more than its fair share. But now, with a neutral monitoring team reviewing fund utilisation, everyone feels more secure. We know that every sector is being held accountable, and that builds trust. Last time, when there were concerns about overlapping expenditures, the monitoring team stepped in and resolved it fairly. It’s refreshing to see such transparency."* ***– Participant 18*** *"Before this system, we often felt like the bigger departments had an advantage—they controlled the funds and never really explained how they were being spent. But with the new reporting process and third-party monitoring, we finally feel like things are being done fairly. Recently, when we flagged a delay in sanitation funding, the reports showed exactly where the bottleneck was, and it was resolved quickly. It’s good to see everyone being held equally accountable."* ***– Participant 10*** |
| CMOC 26 | Smaller sectors are provided with dedicated budgets for capacity-building initiatives. | Training programmes and skill development opportunities for under-resourced departments. | Capacity-building boosts confidence and reduces dependency, enabling these sectors to contribute more actively | Enhanced self-reliance and stronger collaboration in intersectoral interventions. | *"Earlier, we always had to depend on the health department for guidance because we didn’t have the skills or training to handle certain responsibilities on our own. But with the new budget set aside for training, our team has learned how to manage nutrition-focused outreach programmes independently. Last month, we organised a community nutrition drive without needing help from other sectors. It feels good to finally take the lead and show that we’re just as capable."* ***– Participant 6*** |
| CMOC 27 | A new senior officer with a collaborative and supportive leadership style takes charge. | Leadership provides **role clarity, guidance, and effective communication tools** like WhatsApp. | Stakeholders feel **empowered and supported**, perceiving collaboration as essential and progress as attainable. | Improved **collaboration, trust, and responsiveness**, creating a stronger sense of collective purpose. | ***"She brings departments together, reminding everyone that real progress only happens when we work as one. Always just a WhatsApp message away, her responses come quickly." (Participant 13)***  *(***Key words***:* ***guidance****,* ***support****,* ***communication****,* ***progress****).* |
| CMOC 28 | In hierarchical settings, inclusive facilitation during ISC meetings ensures equal participation. | Skilled facilitators, structured agendas, and clear opportunities for all sectors to voice their opinions. | Respectful and inclusive facilitation builds trust, encouraging stakeholders to share their perspectives openly. | Balanced decision-making processes where all sectors feel valued and empowered to contribute. | *Usually, in these meetings, it’s the bigger departments like health or ICDS doing most of the talking, and we just sit quietly, hoping to be heard. But in the last meeting, the facilitator made sure everyone had a turn to speak. They even asked us directly about the issues in our village, which almost never happens. It felt good to know that our perspective actually mattered, and for once, we were part of the decisions being made."* ***– Participant 12*** |
| CMOC 29 | Regular capacity-building sessions help participants feel confident and prepared for ISC meetings. | Tailored training on communication, negotiation, and collaboration for all stakeholders. | Increased confidence enables stakeholders to contribute meaningfully, overcoming psychological barriers. | More inclusive and open discussions, fostering stronger trust and collaboration among sectors. | *"Before these training sessions, I would just sit quietly in meetings, unsure if what I wanted to say even mattered. But after learning how to present my points clearly and confidently, things have changed. In the last meeting, I raised a concern about the lack of educational resources for malnourished children, and for the first time, other departments actually listened and discussed how to support us. It feels like my voice has weight now."* ***– Participant 9*** *"I used to feel intimidated during meetings, especially when health or education dominated the conversations. But the training sessions helped me understand how to articulate our sector’s needs and push for collaboration without hesitation. Last time, I proposed a joint outreach programme for adolescent girls, and it was approved! It’s amazing how a bit of confidence and skill can change how others see you."* ***– Participant 7*** |
| CMOC 30 | Confusion arises in intersectoral meetings when responsibilities are not clearly assigned from the outset. | Clearly defined responsibilities and guidelines are **lacking**, creating **unnecessary extra work**. | Stakeholders feel that **confusion and inefficiency** could be avoided with better-defined roles from the start. | Enhanced **clarity, efficiency, and teamwork** when roles are clearly delineated. | **"We need better-defined responsibilities from the start. If everyone knows their part, there will be less confusion, and there will be no unnecessary extra work, and it will be easier to work together."** (Participant 5) |
| CMOC 31 | Establishing ground rules for respectful communication during meetings. | Agreed-upon rules and norms for respectful engagement, enforced by meeting facilitators. | Respectful communication reduces hierarchical dominance and ensures all voices are acknowledged. | Stakeholders feel valued and included, resulting in equitable contributions and improved collaboration. | *"Before we had these ground rules, meetings were chaotic—people from bigger departments would dominate, and smaller sectors like ours barely got a chance to speak. But now, with clear rules in place, everyone waits their turn, and even disagreements are handled respectfully. Last week, I brought up the need for school-based nutrition awareness, and instead of being sidelined, it was discussed seriously. It feels good to know that my input is respected and contributes to the bigger picture."* ***– Participant 8*** |
| CMOC 32 | Leadership models inclusive behaviour by actively seeking input from all sectors during discussions. | Leadership support for open dialogue, with specific invitations to less vocal sectors to share their views. | Inclusive leadership inspires trust and encourages less powerful stakeholders to share insights without fear. | Collaborative decisions reflect the collective expertise of all sectors, strengthening ISC effectiveness. | *"In most meetings, we’re usually just there to listen while the bigger departments do all the talking. But this time, the block officer specifically asked for our input on the challenges in our village. It was surprising, honestly—it felt like they actually cared about what we had to say. We spoke about the lack of clean water and its impact on children’s health, and they took it seriously. It’s empowering when leadership makes an effort to include everyone, not just the usual voices."* ***– Participant 10*** |
| CMOC 33 | Informal platforms, such as smaller group discussions, are used to encourage shy stakeholders to share ideas. | Breakout sessions and informal platforms like WhatsApp for sharing thoughts outside formal meetings. | Smaller, less intimidating settings help stakeholders overcome hesitancy and share ideas more freely. | Increased participation and engagement, ensuring all sectors contribute to ISC discussions. | *"In those big meetings, it’s hard to speak up—there are so many officials, and you feel like your ideas might not be taken seriously. But during the smaller group discussions, it’s different. Last time, I talked about the need for better sanitation in the village schools, and everyone listened. It’s easier to open up in a smaller setting where you don’t feel judged or overshadowed." – Participant 13*  *"I’ve always found the big meetings a bit overwhelming, especially when health or ICDS dominates the room. But in the breakout sessions, I finally had the space to talk about the gaps in our social protection schemes. The smaller group felt more personal, and my ideas were actually acknowledged. These informal platforms make it so much easier to contribute meaningfully."* ***– Participant 6*** |
| CMOC 34 | Informal gatherings, such as tea breaks or community events, allow participants to connect beyond professional boundaries. | Opportunities for informal interactions outside formal meeting settings. | Personal connections foster mutual respect and reduce hierarchical barriers between sectors. | Stakeholders feel more comfortable collaborating, leading to more cohesive teamwork and resource-sharing. | *"After one of our meetings, we all stayed back for tea, and that’s when the real conversations started. I was talking to someone from health, and we realised that our programmes for school health check-ups weren’t aligned. We ended up brainstorming right there over tea and came up with a plan to coordinate better. It’s moments like these, outside the formal setting, that make us feel like a team instead of just separate departments."* ***– Participant 9*** |
| CMOC 35 | Untied funds are allocated for women’s empowerment and gradually extended to nutrition-related efforts. | Support from **untied funds** and leadership encourages innovative use of resources to address broader needs. | Stakeholders feel **motivated to collaborate** when they see flexibility and shared commitment in resource use. | Expanded **resource utilisation** for intersectoral collaboration, particularly in nutrition programming. | **"Typically, these meetings revolve around utilising untied funds for the betterment of women. What I proposed is that the same funds can also be used to address nutritional deficiencies in children at the household level."** (Participant 3) |
| CMOC 36 | Leadership changes influence meeting dynamics and engagement in intersectoral collaboration (ISC). | Leadership facilitates **meaningful discussions, consistent follow-ups, and problem-solving efforts**. | Stakeholders interpret these meetings as **productive spaces for collaboration and problem resolution**. | Enhanced **engagement and collaboration**, with a shift towards actionable and outcome-driven efforts. | ***"Over the past one and a half years, there’s been a noticeable shift towards meaningful discussions and problem-solving. (Participant 12)"*** *(***Key words***:* ***shift****,* ***meaningful discussions****,* ***consistent follow-up****).* |
| CMOC 37 | Participants use informal communication channels, such as WhatsApp, to maintain regular and candid interactions. | Accessible and user-friendly communication platforms for day-to-day updates and support. | Informal channels enable open, unfiltered communication, strengthening interpersonal relationships. | Improved trust and camaraderie among sectors, leading to quicker decision-making and problem resolution. | *"WhatsApp has been a game changer for us. Whenever there’s an issue, like delays in mid-day meal deliveries, I can immediately message the ICDS or health team. Instead of waiting for formal meetings, we sort things out right away. It’s also helped build better relationships—when you’re constantly in touch, you start seeing each other as teammates rather than just officials from different sectors."* ***– Participant 8*** *"For us in the community, WhatsApp is a lifeline. If there’s a sanitation issue or a nutrition supply delay, I just send a quick message to the officer concerned, and it gets resolved faster than it ever did before. It’s so much easier than formal letters or meetings. And over time, these small interactions have made us trust each other more—it feels like we’re all working toward the same goal."* ***– Participant 12*** |
| CMOC 38 | Cross-sector participants engage in team-building exercises or joint training sessions. | Joint capacity-building initiatives that bring stakeholders together in non-hierarchical settings. | Shared experiences create stronger bonds and reduce the perception of divisions between sectors. | Enhanced sense of shared purpose and more fluid collaboration in implementing health interventions | *"The joint training sessions really changed how we work together. Before, we only knew each other as names on meeting agendas, but during the sessions, we shared stories about the challenges we face in our sectors. It was eye-opening to see how interconnected our work is. Now, when we collaborate, it feels natural because we understand each other’s struggles and strengths. It’s no longer about ‘my sector’ or ‘your sector’—it’s about solving problems together." – Participant 7* |
| CMOC 39 | Leadership encourages informal check-ins between sectoral heads outside of structured meetings. | Regular, informal touchpoints initiated by leadership to build relationships among stakeholders. | Personal interactions beyond formal settings create trust and understanding, strengthening professional bonds. | Collaboration becomes more adaptive and responsive to challenges, with stakeholders feeling valued and connected | *"The block officer encourages us to meet informally to discuss issues. It’s not just about work—it’s about building a partnership. That has really changed how we support each other." (Participant 2, Social Welfare Officer, Dibrugarh)* |
| CMOC 40 | Personal connections foster resource-sharing between departments without relying on formal approvals. | Flexibility in using resources facilitated by trust and goodwill between participants. | Mutual trust and informal relationships reduce bureaucratic delays and encourage a more spontaneous exchange. | Sectors work together seamlessly, enhancing the efficiency and effectiveness of health interventions | *"We recently shared some untied funds with social welfare because they had a pressing need. It wasn’t formalised, but we trusted that they’d use it well—and they did." (Participant 4, Nutrition Officer, Dibrugarh)* |
| CMOC 41 | Collaboration occurs spontaneously based on personal requests rather than formal structures. | Informal **mutual support and flexibility** allow stakeholders to respond to immediate needs. | Stakeholders feel **empowered to act quickly** and **proactively**, without waiting for bureaucratic approvals. | Improved **programme responsiveness** and strengthened interpersonal relationships. | **"Recently I facilitated a session on managing underweight children at a community event without formal invitation just based on the personal request. This spontaneous action was well-received, showcasing the flexibility and mutual support among colleagues."** (Participant 13) |
| CMOC 42 | Roles and responsibilities in intersectoral programmes are not clearly defined in policy documents. | Absence of comprehensive organisational guidelines outlining tasks and scope for each sector. | Ambiguity in roles allows dominant sectors to impose their priorities, sidelining less resourceful sectors. | Increased frustration and disengagement from less powerful sectors, leading to weaker collaboration. | *It’s complete chaos most of the time. We’re called to meetings and asked to ‘collaborate,’ but no one tells us what we’re actually supposed to do. Health just swoops in, takes charge, and leaves us scrambling to figure out how to contribute. Last month, during the nutrition drive, we sat there waiting for instructions, but by the time we were looped in, the health team had already made all the decisions. It’s like we’re extras in a movie where health plays the lead role—and honestly, it’s getting exhausting."* ***– Participant 6*** |
| CMOC 43 | More powerful sectors use their influence to assign additional tasks to less resourced sectors. | Authority and resource disparities that favour larger sectors, with little oversight of task delegation. | Arbitrary task assignments overwhelm smaller sectors, creating resentment and inefficiency. | Participants in weaker positions feel overburdened and undervalued, reducing their ability to collaborate effectively. | *"ICDS is often given extra responsibilities, like community awareness campaigns, without any additional support. It’s unfair because it’s not even part of our main role." (Participant 3, Social Welfare Officer, Dibrugarh)* |
| CMOC 44 | Line departments demonstrate improved attitudes toward intersectoral collaboration due to leadership influence. | Leadership sets **a tone of accountability and inclusivity**, promoting shared responsibility. | Stakeholders feel **valued and motivated**, recognising their roles in collective progress and shared goals. | Strengthened **equitable partnerships** and a deeper commitment to collaboration in ISC initiatives. | ***"This change is gradual and stems from a change in leadership and the attitudes of the line departments involved in ISC." (Participant 12)*** *(***Key words***:* ***leadership influence****,* ***shared responsibility****,* ***attitudes of line departments****).* |
| CMOC 45 | Conflicting directives from multiple levels of leadership exacerbate confusion at the implementation level. | Lack of coordination between district and block leadership results in conflicting messages to programme teams. | Confusion in directives leads to miscommunication and inefficiencies in programme execution. | Stakeholders feel overwhelmed and struggle to align their efforts, reducing the overall effectiveness of programmes. | *"It’s like being caught between two bosses who can’t agree on what they want. The district tells us to focus on one thing, like water supply in schools, and then the block officer says to prioritise sanitation for households. Which one are we supposed to follow? By the time we figure it out, weeks have passed, and nothing actually gets done. It’s so frustrating—how can we make progress when we’re pulled in two different directions****?" – Participant 12*** |
| CMOC 46 | Stakeholders appreciate flexibility in addressing programme needs through informal collaboration. | Informal collaboration builds a sense of **shared purpose and responsibility** among stakeholders. | Stakeholders feel **motivated and valued**, strengthening commitment to collaborative efforts. | Enhanced **mutual support, trust**, and **programme outcomes** through flexible actions. | **"This spontaneous action was well-received, showcasing the flexibility and mutual support among colleagues."** (Participant 13) |
| CMOC 47 | Hierarchical structures create opportunities for dominant sectors to exploit unclear accountability mechanisms. | Power imbalances allow sectors with more resources to deflect responsibilities onto weaker sectors. | Lack of clear accountability mechanisms enables larger sectors to avoid responsibilities, shifting the burden. | Reduced trust and increased conflicts among sectors, undermining effective collaboration. | *"When something doesn’t work, the blame often falls on smaller departments like ours. It feels like we’re always the scapegoat." (Participant 7, Community Representative, Dibrugarh)* |
| CMOC 48 | Lack of clarity in operational policies causes duplication of tasks across sectors. | Overlapping mandates and inadequate policy frameworks for defining sectoral roles and responsibilities. | Duplication of tasks creates inefficiency, as sectors repeat efforts instead of working collaboratively. | Resource wastage and reduced efficiency in programme implementation | *"It’s honestly a mess sometimes. We’ll be running a hygiene awareness campaign in schools, only to find out that public health engineering has already done the same thing a week before. No one tells us who is handling what, and we just keep overlapping efforts. It’s not just frustrating—it’s a total waste of time and resources. If we actually coordinated properly, we could achieve so much more instead of repeating the same work****." – Participant 3*** |
| CMOC 49 | The Rural Livelihood Mission provides platforms for community meetings and women-centric initiatives. | **Community meeting platforms** and **untied funds** are shared by the livelihood sector to facilitate collaboration. | Stakeholders feel **encouraged and empowered** to use shared resources for addressing cross-sectoral issues like nutrition. | Improved **resource-sharing practices** and integration of sectoral goals, particularly for nutrition outcomes. | **"The livelihood mission in my block provides us with great community meeting platforms. Our field staff often organise women-centric awareness campaigns there and even invite our experts for sessions sometimes, which is really encouraging to see."** (Participant 3) |
| CMOC 50 | Unclear communication channels lead to confusion about reporting structures and decision-making authority. | Lack of formalised communication protocols and reporting frameworks across sectors. | Confusion about who to report to or seek approvals from creates delays and inefficiencies in task execution. | Stakeholders feel frustrated and demotivated, further weakening intersectoral collaboration. | *"We are often told to submit reports, but it’s unclear who needs them or how they are being used. It just adds to the confusion." (Participant 8, Nutrition Officer, Dibrugarh)* |
| CMOC 51 | Certain sectors, such as the Rural Livelihood Mission, receive higher funding and have more staff than others. | Uneven **budget allocations** and **staff strength** among sectors create resource disparities. | Less-resourced sectors perceive **dependency** on better-funded sectors, which exacerbates power imbalances. | **Perceived power imbalances** and strained collaboration among sectors. | **"The Rural Livelihood Mission tends to receive more funding compared to other sectors, with health following closely behind. The Rural Livelihood Mission also has a larger staff presence."** (Participant 8) |
| CMOC 52 | Arbitrary task assignment in hierarchical settings increases resentment among field-level workers. | Tasks assigned without consultation or consideration of staff capacities at the field level. | Staff feel their input is ignored, leading to demotivation and a lack of ownership over their work. | Poor morale among field workers reduces the quality of programme implementation and collaboration. | *"Tasks are often handed down to us without asking if we have the capacity to handle them. It feels like we’re being overworked with no say in the matter." (Participant 9, Field Worker, Dibrugarh)* |
| CMOC 53 | Dominant sector claims credit for collaborative work. | No attribution agreements. | Resentment undermines future cooperation. | Withdrawal of effort by marginalised sectors. | *“It’s always the same story—everyone works together, but when it’s time to showcase the results, only the health department gets the credit. We put in just as much effort, sometimes even more, but when reports are written or presentations are made, our contributions are barely mentioned. It’s frustrating! Why should we keep putting in the work when we know we’ll just be sidelined in the end? If this keeps happening, people will stop bothering to collaborate at all."* ***– Participant 7*** |
| CMOC 54 | Mentorship pairs from different sectors. | Mutual learning builds empathy and respect. | Shared problem-solving approaches. | Shared problem-solving approaches. | *"I was paired with a mentor from the health department, and honestly, I never realised how complex their workload was. Before, we used to think they were just ignoring our requests, but now I see the pressures they deal with. It’s completely changed how I approach collaboration. Instead of blaming each other, we actually sit down and find solutions together. This kind of mentorship should have started years ago!" – Participant 5  "My mentor is from Health, and I had no idea how much their work impacts school health. Before, I just assumed they handled infrastructure, but now I see how sanitation, water supply, and even menstrual hygiene tie directly into student well-being. Working together has made planning so much smoother—we’re not just two departments anymore, we’re a team solving the same problems from different angles."* ***– Participant 9*** |
| CMOC 55 | Joint community based awareness programmes. | Volunteering days. | Shared goals foster camaraderie. | Increased informal communication. | *“During POSHAN Maah, we didn’t just sit in meetings and talk about nutrition—we actually got out into the community. One day, we organised a village cooking demonstration where health, ICDS, and Education teams worked together to show mothers how to prepare nutritious meals with locally available ingredients. It was so much more effective than just giving lectures! Working side by side made us feel like a real team, not just officials from different departments."* ***– Participant 10***  *"This year’s POSHAN Maah felt different because we actually did things together. Instead of the usual presentations, we had a community clean-up and nutrition drive where all sectors joined in—health workers, ASHAs, schoolteachers, even local leaders. As we worked, we had real conversations—not just about policies, but about the actual challenges families face. It brought us closer, and honestly, it did more for collaboration than any formal meeting ever could."* ***– Participant 6*** |
| CMOC 56 | Annual cross-sector retreats. | Offsite venues. | Shared experiences create lasting bonds. | Resilient partnerships during crises. | *"I’ll admit, when they first announced the cross-sector retreat, I thought it was just another bureaucratic exercise. But once we got there, away from the usual office setting, it was completely different. We shared meals, played team games, and actually got to know each other as people, not just job titles. Now, when a crisis comes up, I don’t hesitate to call my counterparts in ICDS or NGO because I know them beyond just meetings—we’ve built real trust."* ***– Participant 3*** *"At the retreat, we had this silly team-building exercise where we had to solve problems together—it was frustrating, hilarious, and honestly, one of the best things we’ve done. It broke the usual barriers between departments. Now, when we face real challenges, we don’t waste time arguing over whose responsibility it is. We’ve already learned how to work together, and that makes all the difference when things get tough."* ***– Participant 14*** |
| CMOC 57 | Policy drafts are co-signed by all sectors. | Joint accountability agreements. | Collective ownership fosters pride in outcomes. | High adherence to policy implementation. | *"When we used to get policies, it always felt like they were just handed down to us, with no real say in shaping them. But this time, when we saw all our department logos side by side on the final POSHAN Maah action plan, it felt different—like we all had a stake in making it work. It wasn’t just a health or ICDS document anymore; it was ours. That sense of ownership has made everyone more committed to actually implementing it." – Participant 7* |
| CMOC 58 | Needs-assessment tools allocate resources. | Data-driven allocation algorithms. | Reduces subjective bias in funding. | Perceived fairness across sectors. | *"Before, funding decisions felt like they were based more on who had the right connections rather than actual need. But now, with data-driven tools, there’s no room for favoritism. Last time, when we allocated nutrition funds, we used real malnutrition data instead of just relying on department preferences. It was the first time I saw every sector accept the decision without any complaints—because the numbers made it fair."* ***– Participant 15*** *"I can’t tell you how many times we’ve lost out on funding simply because bigger sectors had more influence in decision-making. But now, with needs-assessment tools, the process is transparent. During the last budget cycle, our request for additional staff got approved—not because we pushed for it, but because the data showed the gap clearly. It’s a relief to finally see resources going where they’re actually needed, not just where the loudest voices are."* ***– Participant 5*** |
| CMOC 59 | In hierarchical settings, certain sectors, like the health department, dominate discussions in intersectoral meetings. | The **authority and influence** of more powerful sectors shape the agenda and dominate decision-making. | Stakeholders from less powerful sectors feel **ignored and undervalued**, leading them to withhold their input. | Poor **engagement** and **limited participation**, reducing the effectiveness of collaborative actions. | **"The senior officer from the health department takes the floor more often, wielding a wealth of information that subtly commands attention. His words carry weight, and, more often than not, it’s his points that make it into the official record."** (Participant 14) |
| CMOC 60 | Stakeholders perceive a lack of clear agenda or inclusive representation in convergence meetings. | The absence of **structured agendas** that incorporate all departments’ indicators creates confusion. | Stakeholders feel **excluded and less motivated** to engage meaningfully in the discussion. | Weak **coordination and collaboration** among sectors during intersectoral meetings. | **"In convergence meetings, the topics of discussions should be fixed, incorporating criteria from all the departments. All departments should be aware of each other’s indicators; if this happens from the state level, it would be much better."** (Participant 5) |
| CMOC 61 | Current meeting structures fail to ensure fair participation or represent underrepresented groups like women members | Lack of mechanisms for **equal representation** and **inclusion of community voices** weakens engagement. | Stakeholders feel **disempowered**, with limited authority to push for meaningful changes in decisions. | Inequitable **representation and participation** lead to poor trust and diminished collaboration. | **"There should be fair participation from all the sectors. Meetings should also involve women members of the community, not just public representative members."** (Participant 16) |
| CMOC 62 | Stakeholders engage in informal conversations to discuss unresolved or unaddressed issues from formal meetings. | Informal settings allow for **spontaneous problem-solving and idea exchange** outside structured meetings. | Stakeholders feel **enabled to share ideas and resolve issues informally**, fostering more effective collaboration. | Enhanced **teamwork and programme alignment** due to shared understanding and informal coordination. | **"We do meet often over tea and discuss a lot many things which we don’t discuss in meetings, since we know each other well it’s easier to work together more closely."** (Participant 14) |
