# Supplementary material for: Understanding the influence of power dynamics in intersectoral collaboration: A realist evaluation in Assam, India
Source: PLOS Glob Public Health. 2025 Dec 12;5(12):e0005639. doi: 10.1371/journal.pgph.0005639 (PMC12700422; doi:10.1371/journal.pgph.0005639)
Supplement: S3 Text — (DOCX) [file pgph.0005639.s003.docx]

#### **Supplementary file 3** : PT refinement process for PT 2 to PT 6

| PT 2 | When leadership structures are democratic, this creates an environment that values inclusivity, participation, shared decision-making, and transparent communication is evident. This environment inspires commitment and empowers individuals by providing them with resources and autonomy, thereby reinforcing their collective belief in achieving shared goals. This fosters equitable partnerships, counter power imbalances, and strengthen connections, ultimately enhancing collaboration in intersectoral interventions. | | | | |
| --- | --- | --- | --- | --- | --- |
| **CMOC No** | **Context** | **Mechanism** | | **Outcome** | **Supporting quote** |
|  |  | **Resource** | **Reasoning** |  |  |
| 12 | A new senior officer with a collaborative and supportive leadership style takes charge. | Leadership provides **role clarity, guidance, and effective communication tools** like WhatsApp. | Stakeholders feel **empowered and supported**, perceiving collaboration as essential and progress as attainable. | Improved **collaboration, trust, and responsiveness**, creating a stronger sense of collective purpose. | **"She brings departments together, reminding everyone that real progress only happens when we work as one. Always just a WhatsApp message away, her responses come quickly." (Participant 13)** *(*Key words*:* **guidance***,* **support***,* **communication***,* **progress***).* |
| 13 | Leadership changes influence meeting dynamics and engagement in intersectoral collaboration. | Leadership facilitates **meaningful discussions, consistent follow-ups, and problem-solving efforts**. | Stakeholders interpret these meetings as **productive spaces for collaboration and problem resolution**. | Enhanced **engagement and collaboration**, with a shift towards actionable and outcome-driven efforts. | **"Over the past one and a half years, there’s been a noticeable shift towards meaningful discussions and problem-solving. (Participant 12)"** *(*Key words*:* **shift***,* **meaningful discussions***,* **consistent follow-up***).* |
| 14 | Line departments demonstrate improved attitudes toward intersectoral collaboration due to leadership influence. | Leadership sets **a tone of accountability and inclusivity**, promoting shared responsibility. | Stakeholders feel **valued and motivated**, recognising their roles in collective progress and shared goals. | Strengthened **equitable partnerships** and a deeper commitment to collaboration in ISC initiatives. | **"This change is gradual and stems from a change in leadership and the attitudes of the line departments involved in ISC." (Participant 12)** *(*Key words*:* **leadership influence***,* **shared responsibility***,* **attitudes of line departments***).* |
| 15 | Leadership models inclusive behaviour by actively seeking input from all sectors during discussions. | Leadership support for open dialogue, with specific invitations to less vocal sectors to share their views. | Inclusive leadership inspires trust and encourages less powerful stakeholders to share insights without fear. | Collaborative decisions reflect the collective expertise of all sectors, strengthening ISC effectiveness. | *"In most meetings, we’re usually just there to listen while the bigger departments do all the talking. But this time, the block officer specifically asked for our input on the challenges in our village. It was surprising, honestly, it felt like they actually cared about what we had to say. We spoke about the lack of clean water and its impact on children’s health, and they took it seriously. It’s empowering when leadership makes an effort to include everyone, not just the usual voices."* **– Participant 10** |
| 16 | Leadership ensures that additional funds for nutrition initiatives are shared across all sectors. | Transparent processes for resource-sharing and equal access to financial allocations. | Clear and open distribution mechanisms reduce resentment and foster inclusivity among stakeholders. | Better participation from all sectors, reducing dependency and enhancing mutual respect in collaboration. | *"Before, it always felt like the health department controlled everything, and we just had to adjust with whatever little was left. But now, with BDO mam, we actually get a say in how funds are used. This time, we were able to allocate resources specifically for social protection schemes that support malnourished children beyond just medical treatment. Participant 4   "In our village meetings, people used to ask why only health camps were getting support while other basic needs, like behaviour change campaigns, or employment recruitment drives were ignored. Now that funds are shared more equally, we can do at least once In a month different camps. " Participant 12* |
| 17 | Convergence meetings focus on specific, actionable topics to maintain engagement across sectors. | Well-structured agendas, action points, and frequent follow-ups by leadership. | Focused discussions ensure stakeholders stay engaged and see the relevance of their contributions. | Increased stakeholder participation and more actionable outcomes from collaborative efforts. | *"I remember how frustrating these meetings used to be, endless discussions, everyone talking in circles, and in the end, no real decisions. It felt like we were attending meetings just for the sake of it. But things have changed now. The DSWO sir makes sure there’s a clear agenda, and every sector knows what they need to contribute. We come in, discuss what actually matters, and leave with action points instead of just ideas floating in the air. Last month, we had a convergence meeting on school nutrition, and instead of just hearing updates, we broke into small groups, set priorities, and assigned responsibilities on the spot. Within a week, I saw follow-ups happening, schools getting water filters repaired, ICDS linking mid-day meals with health check-ups. When meetings are structured well, people actually feel like their time is valued, and things get done."*  *– Participant 9*  *"Before, these meetings felt like a formality, just long discussions where nothing really changed on the ground. But now, with proper agendas and clear follow-ups, we actually see results., meetings where problems are actually solved, not just discussed."* |
| 18 | Hierarchical systems, Health departments dominate discussions in intersectoral meetings due to their perceived authority. | Meeting platforms, leadership from health officials, and budgetary control. | The dominance of the health sector discourages other departments from voicing their opinions. | Reduced participation from less powerful sectors, leading to imbalanced collaboration. | *"Whenever we have a meeting, it always feels like the health department has the final word. No matter what we from WASH or ICDS say, it’s like our ideas don’t really count. It’s frustrating, you know? After a while, it makes us feel like there’s no point in speaking up because no one’s really listening. It’s hard to stay motivated when it feels like only one side matters in the end." -Participant 3* |
| 19 | Hierarchal meeting settings/ meeting led by senior officials but designed to include inputs from all sectors. | Structured **agendas and follow-ups** ensure clear roles and responsibilities for all stakeholders. | Stakeholders experience **role clarity** and feel their contributions are essential to the programme's success. | Enhanced **coordination and shared ownership**, fostering a balanced environment. | **"Frequent meetings with clearly defined roles and follow-ups have helped clarify our responsibilities and fostered mutual respect among departments. This makes collaboration more meaningful."** *(Participant 11)  "These committees have given us a space to talk about the real problems in our villages. Before, it always felt like decisions were made far away, without any input from the community. But now, we’re invited to these meetings, and they ask for our perspectives. Last time, I raised concerns about malnourished children not getting regular Anganwadi rations, and the issue was taken seriously. It’s empowering to know that what we say actually matters." (Participant 4)*  *(*Key words*:* **roles***,* **responsibilities***,* **mutual respect***,* **meaningful***).* |
| 20 | In hierarchical settings, inclusive facilitation during ISC meetings ensures equal participation. | Skilled facilitators, structured agendas, and clear opportunities for all sectors to voice their opinions. | Respectful and inclusive facilitation builds trust, encouraging stakeholders to share their perspectives openly. | Balanced decision-making processes where all sectors feel valued and empowered to contribute. | *Usually, in these meetings, it’s the bigger departments like health or ICDS doing most of the talking, and we just sit quietly, hoping to be heard. But in the last meeting, the facilitator made sure everyone had a turn to speak. They even asked us directly about the issues in our village, which almost never happens. It felt good to know that our perspective actually mattered, and for once, we were part of the decisions being made."* **– Participant 12** |
| 21 | Establishing ground rules for respectful communication during meetings. | Agreed-upon rules and norms for respectful engagement, enforced by meeting facilitators. | Respectful communication reduces hierarchical dominance and ensures all voices are acknowledged. | Stakeholders feel valued and included, resulting in equitable contributions and improved collaboration. | *"Before we had these ground rules, meetings were chaotic, people from bigger departments would dominate, and smaller sectors like ours barely got a chance to speak. But now, with clear rules in place, everyone waits their turn, and even disagreements are handled respectfully. Last week, I brought up the need for school-based nutrition awareness, and instead of being sidelined, it was discussed seriously. It feels good to know that my input is respected and contributes to the bigger picture."* **– Participant 8** |
| 22 | Stakeholders perceive a lack of clear agenda or inclusive representation in convergence meetings. | The absence of **structured agendas** that incorporate all departments’ indicators creates confusion. | Stakeholders feel **excluded and less motivated** to engage meaningfully in the discussion. | Weak **coordination and collaboration** among sectors during intersectoral meetings. | **"In meetings, the topics of discussions should be fixed, incorporating criteria from all the departments. All departments should be aware of each other’s indicators; if this happens from the state level, it would be much better."** *(*Participant 5*)* |
| Thought process | The PT broadly focused on how democratic leadership fosters inclusivity, participation, and transparent communication. While it captured the ideal qualities of effective leadership, it lacked specificity in addressing the unique challenges posed by **hierarchical health systems**. The refinement process contextualised these leadership qualities within the constraints of hierarchical structures, where power imbalances and rigid authority lines often impede collaboration.  For example, the participant quotes revealed that leadership actions like **clarifying roles and responsibilities**, **creating communication channels**, and **aligning sector goals** play a critical role in reducing confusion and fostering a sense of purpose. The PT thus evolved to describe not just the broad principles of democratic leadership, but how leadership operates in **hierarchical contexts** to create environments that empower staff, reduce dominance, and enable meaningful collaboration.  **Key Change**: The PT explicitly situates leadership within hierarchical health systems, specifying mechanisms like role clarity, goal alignment, and communication that create a supportive and empowering environment.  **Emphasising Power Dynamics and Equity**  While the PT mentioned inclusivity and empowerment, it did not fully explore **how leadership could address and mitigate power imbalances** in hierarchical systems. The refinement process explicitly focused on this, informed by evidence from the CMOCs. For instance, participant quotes highlighted how **leadership attitudes** and consistent follow-ups led to equitable participation and shared responsibility among sectors.  The refined PT emphasised how leadership actions, such as transparent communication and inclusive decision-making, help flatten hierarchies and **redistribute power**. For example, the quote about a senior officer providing timely responses and fostering collaboration illustrated how leadership behaviour can create an equitable and motivating environment.  **Key Change**: The refined PT highlights the role of leadership in actively breaking down hierarchical barriers, reducing power imbalances, and creating a sense of equity and shared responsibility among stakeholders.  **Formal and Informal Mechanisms**  The PT alluded to inclusivity and transparency but did not specify **how leadership creates these conditions**. The refined PT incorporates evidence from the CMOCs to emphasise the importance of both **formal mechanisms** (e.g., structured meetings, clear agendas, and follow-ups) and **informal mechanisms** (e.g., interpersonal communication, trust-building). For example, participant quotes about WhatsApp communication and consistent leadership support show how informal mechanisms can complement formal structures to create a cohesive and engaged team.  By including both types of mechanisms, the PT demonstrates how leadership combines structure with flexibility to foster a collaborative environment. This dual approach ensures that leadership efforts are grounded in both procedural consistency and relational trust.  **Key Change**: The refined PT adds the dual role of **formal and informal mechanisms**, such as structured task forces and interpersonal communication, in creating a supportive and inclusive leadership environment.  Summary: The refinement of the PT into the PT involved contextualising leadership actions within hierarchical systems, explicitly addressing power dynamics and equity, and incorporating both formal and informal mechanisms. The revised PT is evidence-based, aligning with participant quotes and CMOCs to provide a nuanced understanding of how leadership drives empowerment, trust, and collaboration while minimising hierarchical barriers. | | | | |
| Mechanism Elaboration: Triggers | Key Components Added to refine PT2  To move beyond broad descriptors like “democratic leadership,” PT2 now specifies four sub-mechanisms derived from participant data:  Transparent Communication  *Trigger*: Leaders actively share information (e.g., WhatsApp updates, open meeting minutes) and maintain accessibility.  *Participant Evidence*: *“Her responses come quickly, making her support feel like a steady presence.”* (Participant 13)  Participatory Agenda-Setting  *Trigger*: Collaborative forums co-design meeting agendas (e.g., pre-meeting surveys, rotating sector leads).  *Participant Evidence*: *“Discussions are now aligned with action plans that include diverse perspectives.”* (Participant 13)  Resource Autonomy  *Trigger*: Departments gain control over discretionary funds or personnel to implement decisions.  *Participant Evidence*: *“Real progress only happens when we work as one.”* (Participant 13)  Facilitative Accountability  *Trigger*: Leaders enforce follow-ups (e.g., progress trackers) while supporting problem-solving.  *Participant Evidence*: *“Consistent follow-up and concrete actions make this shift ingrained.”* (Participant 12) | | | | |
| Boundary conditions | PT2’s effectiveness depends on four contextual factors:  Leadership Continuity: Democratic practices must persist beyond individual leaders (e.g., institutionalised via succession planning).  Cultural Legitimacy of Participation: Stakeholders must view inclusive norms as credible (not “tokenism”).  Resource Decentralisation: Genuine autonomy requires fiscal/personnel authority beyond rhetoric.  Time for Trust-Building: Outcomes like *collective efficacy* emerge gradually (Participant 12: *“This change is gradual…”*). | | | | |
| Temporal dimension | PT2 now acknowledges that outcomes unfold in three phases:  Early Phase: Scepticism (*“meetings felt repetitive”*) due to legacy of hierarchical norms.  Middle Phase: Trust-building via consistent mechanisms (e.g., WhatsApp responsiveness, co-designed agendas).  Mature Phase: Internalisation of collective efficacy (*“collaboration feels essential”*). | | | | |
| Refined PT 2 | "In hierarchical health system structures, when leadership actively supports **open communication**, aligns **sector goals with sectoral priorities**, and ensures **appropriate resource allocation**, it creates a **supportive environment** that motivates, **empowers**, and engages staff. This **leadership-driven approach** fosters **team efficacy** and **shared responsibility**, as staff members feel **valued**, **supported**, and confident in their ability to contribute meaningfully. Empowered by leadership, staff are more likely to take **ownership of their roles**, contribute **proactively**, and trust in their team's collective capabilities. **Transparent communication structures** help break down **hierarchical barriers**, reduce **power imbalances**, and foster a sense of **equity** within the team. By creating a culture of **openness** and **inclusivity**, leadership promotes a **collaborative environment** where **power dynamics are minimised**, and staff are encouraged to work together towards common goals." | | | | |
| *PT 2 (Simplified)* | *Refined Context-Mechanism-Outcome (CMO) Statement  In hierarchical governance systems where intersectoral collaboration is traditionally constrained by centralised authority and passive participation norms (Context), the consistent practice of democratic leadership, characterised by transparent communication, participatory agenda-setting, resource autonomy, and facilitative accountability (Mechanisms), reconfigures power dynamics by legitimising marginalised voices, redistributing influence, and aligning sectoral priorities. This fosters equitable partnerships, sustained commitment, and collective efficacy in achieving shared goals (Outcomes).* | | | | |

| PT 3 | When resources are unevenly or inadequately distributed among sectors, it increases the dependency of less influential or less powerful sectors and exacerbates perceived power imbalances. This inequitable resource allocation not only hampers sectoral engagement and collaborative action in programme implementation but also undermines the sustainability of the partnership, as it fosters less enduring and more inequitable relationships among the collaborating sectors. | | | | |
| --- | --- | --- | --- | --- | --- |
| CMOC No | Context | Mechanism | | Outcome | Supporting quote |
|  |  | Resource | Reasoning |  |  |
| 23 | Certain sectors, such as the Rural Livelihood Mission, receive higher funding and have more staff than others. | Uneven **budget allocations** and **staff strength** among sectors create resource disparities. | Less-resourced sectors perceive **dependency** on better-funded sectors, which exacerbates power imbalances. | **Perceived power imbalances** and strained collaboration among sectors. | **"The Rural Livelihood Mission tends to receive more funding compared to other sectors, with health following closely behind. The Rural Livelihood Mission also has a larger staff presence."** (Participant 8) |
| 24 | The Rural Livelihood Mission provides platforms for community meetings and women-centric initiatives. | **Community meeting platforms** and **untied funds** are shared by the livelihood sector to facilitate collaboration. | Stakeholders feel **encouraged and empowered** to use shared resources for addressing cross-sectoral issues like nutrition. | Improved **resource-sharing practices** and integration of sectoral goals, particularly for nutrition outcomes. | **"The livelihood mission in my block provides us with great community meeting platforms. Our field staff often organise women-centric awareness campaigns there and even invite our experts for sessions sometimes, which is really encouraging to see."** (Participant 3) |
| 25 | Untied funds are allocated for women’s empowerment and gradually extended to nutrition-related efforts. | Support from **untied funds** and leadership encourages innovative use of resources to address broader needs. | Stakeholders feel **motivated to collaborate** when they see flexibility and shared commitment in resource use. | Expanded **resource utilisation** for intersectoral collaboration, particularly in nutrition programming. | **"Typically, these meetings revolve around utilising untied funds for the betterment of women. What I proposed is that the same funds can also be used to address nutritional deficiencies in children at the household level."** (Participant 3) |
| 26 | Cross-sectoral resource sharing is supported by leadership. | Leadership fosters **collaborative decision-making** by encouraging flexible use of resources for shared goals. | Stakeholders feel **valued and supported**, strengthening their commitment to collaboration. | Strengthened **intersectoral partnerships** and improved programme sustainability. | **"Our Chief Executive Officer sir has been supportive of this initiative, which is fantastic. I believe that resource sharing is crucial if we want to see positive outcomes, regardless of the department/sector involved."** (Participant 3) |
| 27 | Equitable distribution of untied funds across sectors at the block level. | Untied funds allocated proportionately to all sectors, enabling each to address its specific needs | Stakeholders feel their needs are recognised, fostering trust and fairness in collaboration. | Increased autonomy of less-resourced sectors, leading to improved collaboration and reduced power imbalances. | *"For the longest time, we had to rely on whatever was left after the bigger departments took their share, and honestly, it felt like we were just tagging along. But now, at least some part of untied funds being distributed fairly, we finally have the freedom to plan according to our actual needs.*  *It’s a big shift. Public administration also benefits because it no longer has to keep mediating between sectors fighting over resources. When funding is fair, collaboration becomes smoother, no more back-and-forth delays or departments feeling sidelined." Participant 7* |
| 28 | Joint procurement of essential supplies for nutrition and WASH programmes at the block level. | Shared procurement systems that meet the needs of all sectors, with equal representation in decision-making. | Collaborative resource management ensures smaller sectors are not overshadowed, promoting shared ownership. | Enhanced collaboration and trust, with a collective sense of accountability for resource use. | *"Earlier, procurement was a nightmare, each sector fighting for their own supplies, delays in approvals, and endless paperwork. Health would often get priority, while education and ICDS had to wait. But now, with joint procurement, few thongs streamlined. – Participant 3  "In the past, WASH struggled to get enough sanitation kits for schools, while the health department had extra medical supplies that weren’t immediately used. Now that procurement is planned together, we can make sure every sector gets what’s needed without wastage.***– Participant 13** |
| 29 | Decentralised resource management ensures funds are allocated based on the specific needs of sectors. | Local-level input in budget decisions and resource allocation based on block-level priorities. | Decentralised decision-making empowers sectors to identify and address their own priorities without external bias. | Increased efficiency in resource use, improved collaboration, and reduced power hierarchies. | *"Before, most budget decisions were made in Guwahati, and by the time the funds reached Dibrugarh, they were already earmarked for things that didn’t always match local needs. Now that decisions are happening at the block level, we finally have the flexibility to allocate funds where they’re actually needed.." – Participant 15* |
| 30 | Continuous coordination and collaboration are required to keep intersectoral processes functional | Over time, staff develop an **understanding of the importance of coordination** through lived experience. | Stakeholders feel **motivated to engage in coordination efforts**, despite the lack of clear guidelines. | Improved **interpersonal relationships** but at the cost of **efficiency** and **overwork**. | **"Major work was to keep the convergence alive. I slowly realised the importance of continuous coordination and collaboration with various stakeholders."** *(*Participant 6) |
| 31 | Equitable distribution of field staff across all programme areas. | Additional staff positions created to reduce workload disparities between sectors. | Balanced staffing reduces over-reliance on certain departments and ensures equal participation in interventions. | Improved programme implementation and more meaningful involvement of all sectors. | *"In the past, we used to see health staff everywhere, while other departments like social welfare and academia barely had anyone on the ground. It felt like everything was falling on health, and the other sectors were just struggling to catch up. But now, with more staff hired across all departments, things are much more balanced. ICDS workers are able to handle their own responsibilities, like nutrition distribution, without constantly relying on health staff. It’s made the work smoother, and we’re seeing better coordination because everyone is pulling their weight."* **– Participant 11** |
| 32 | Stakeholders appreciate flexibility in addressing programme needs through informal collaboration. | Informal collaboration builds a sense of **shared purpose and responsibility** among stakeholders. | Stakeholders feel **motivated and valued**, strengthening commitment to collaborative efforts. | Enhanced **mutual support, trust**, and **programme outcomes** through flexible actions. | **"This spontaneous action was well-received, showcasing the flexibility and mutual support among colleagues."** *(*Participant 13*)* |
| Thought Process | **Expanding the PT: Moving from General to Specific**  The PT broadly acknowledged that inequitable resource allocation among sectors creates dependency, limits collaboration, and exacerbates power imbalances. However, it lacked specificity in describing how **resource sharing mechanisms** can promote equity and strengthen collaboration.  The revised PT contextualises this dynamic by situating it within **low-resource settings**, where inequitable resource allocation has tangible implications for intersectoral collaboration. Insights from the CMOCs helped illustrate how shared resources, like **untied funds** and **community platforms**, can serve as practical tools for balancing resource access across sectors. For example, Participant 3 described how livelihood associations began using untied funds for child nutrition, demonstrating how shared resources can address cross-sectoral goals.  **Key Change**: The revised PT explicitly emphasises **equitable resource allocation** and its role in empowering less resource-endowed sectors, making the theory more actionable and grounded in real-world practices.  **Emphasising the Role of Power Dynamics and Stakeholder Empowerment**  The PT recognised resource inequities but did not fully explore how **equity in resource allocation impacts power hierarchies**. The revised PT explicitly addresses how equitable resource sharing diminishes dependency on dominant sectors and fosters a sense of **autonomy and self-reliance** among less powerful sectors.  For instance, Participant 8 highlighted how certain sectors, like the Rural Livelihood Mission, are resource-rich compared to others. This observation informed the revised PT’s focus on **reducing reliance on traditionally dominant sectors** by ensuring fair resource allocation. Furthermore, Participant 3’s account of supportive leadership and resource-sharing efforts illustrated how inclusive practices create an environment where all sectors feel valued and capable of meaningful contributions.  **Key Change**: The revised PT integrates **stakeholder empowerment** as a central mechanism, showing how fair resource distribution helps balance power dynamics and fosters **autonomy**, leading to more equitable partnerships.  **Formal and Informal Mechanisms**  The PT referred to resource distribution as a static concept but did not account for the **mechanisms** that enable equitable resource sharing. The revised PT incorporates both **formal mechanisms** (e.g., leadership support for untied funds and decision-making structures) and **informal mechanisms** (e.g., collaboration through community meeting platforms).  For example, Participant 3 described how informal settings, like women-centric campaigns and livelihood platforms, provided opportunities for cross-sectoral collaboration. These informal spaces were complemented by leadership support, which served as a formal mechanism to institutionalise resource sharing. This combination of formal and informal approaches allowed stakeholders to leverage resources effectively while feeling supported in their collaborative efforts.  **Key Change**: The revised PT highlights the dual role of **formal mechanisms** (e.g., untied funds, leadership) and **informal mechanisms** (e.g., community platforms, relational trust) in enabling equitable resource allocation and fostering collaboration.  The PT mentioned the negative effects of inequitable resource allocation but did not clearly articulate how **mechanisms like fairness and autonomy** translate into **better collaboration and reduced power imbalances**. The revised PT builds on the CMOCs to strengthen these causal links.  For instance, the CMOCs illustrated that equitable resource allocation creates a sense of **fairness and inclusivity**, motivating sectors to engage more actively. Participants described how flexible resource use and shared goals empowered them to contribute meaningfully, even in the face of systemic disparities. This led to stronger collaboration and a more egalitarian environment where traditional power hierarchies held less influence.  **Key Change**: The revised PT explicitly connects equitable resource allocation to outcomes like **reduced dependency**, **greater autonomy**, and **enhanced collaboration**, making the causal pathways clearer and more robust.  **Summary**  The refinement of the PT into the revised PT involved:  **Contextualising resource dynamics** within low-resource settings to emphasise the practical importance of equity in resource allocation.  **Highlighting empowerment and autonomy** as key outcomes of fair resource distribution, showing how these mechanisms address power imbalances.  Incorporating **formal and informal mechanisms**, such as leadership-driven resource sharing and community platforms, to demonstrate how equity can be operationalised.  Strengthening **causal links** between mechanisms and outcomes, showing how fairness and inclusivity lead to reduced power imbalances and stronger collaboration.  The revised PT is grounded in evidence from the CMOCs and participant experiences, offering a detailed and actionable framework for addressing resource inequities and fostering collaboration in intersectoral interventions. This iteration moves beyond the general acknowledgment of resource imbalances to provide practical pathways for reducing dependency and creating equitable partnerships. | | | | |
| Mechanism Elaboration: Triggers | Key Components Added to refine PT 3  PT3 now specifies four sub-mechanisms, grounded in participant data:  Decentralised Resource Control  *Trigger*: Allowing sectors autonomy over flexible funds (e.g., "untied funds" for nutrition initiatives).  *Participant Evidence*: *“A few livelihood associations have started using untied funds for their children’s nutrition… Our CEO has been supportive.”* (Participant 3)  Cross-Sectoral Resource Pooling  *Trigger*: Formal agreements to share platforms (e.g., community meetings) or personnel.  *Participant Evidence*: *“The livelihood mission provides community meeting platforms… Our field staff organise campaigns there and invite health experts.”* (Participant 3)  Participatory Resource Prioritisation  *Trigger*: Joint decision-making on resource use (e.g., health workers proposing nutrition-focused fund allocation).  *Participant Evidence*: *“I proposed that untied funds can address nutritional deficiencies… Resource sharing is crucial for outcomes.”* (Participant 3)  Transparency in Resource Allocation  *Trigger*: Public disclosure of budgets (e.g., Rural Livelihood Mission vs. health sector funding).  *Participant Evidence*: *“The Rural Livelihood Mission receives more funding… Health has a slight advantage in qualified personnel.”* (Participant 8) | | | | |
| Boundary conditions | PT3 operates effectively only when:  Leadership Champions Redistribution: Supportive figures (e.g., Participant 3’s CEO) legitimise resource-sharing norms.  Flexible Funding Structures Exist: Resources must be re-allocatable (e.g., untied funds).  Community Platforms Are Accessible: Shared spaces (e.g., livelihood mission’s meetings) enable cross-sector dialogue.  Powerholder Buy-In: Dominant sectors (e.g., Rural Livelihood Mission) agree to cede control. | | | | |
| Temporal dimension | PT3’s outcomes unfold in three phases:  Resistance Phase: Dominant sectors resist sharing (e.g., Participant 8’s budget hierarchy).  Experimentation Phase: Early wins (e.g., Participant 3’s nutrition-focused fund use) build trust.  Institutionalisation Phase: Resource-sharing becomes routine (e.g., “CEO support” normalises the practice). | | | | |
| Refined PT 3 | "In a low-resource setting, when **resources are distributed equitably** and made **accessible to all sectors**, it **mitigates the dependency** of less resource-endowed sectors on more powerful ones. This **equitable distribution** fosters a sense of **fairness** and **inclusivity** among stakeholders, empowering all sectors to participate **more actively** and **meaningfully** in collaborative processes. By **reducing disparities in resource allocation**, participants from less powerful sectors feel **valued** and **capable**, which fosters **autonomy** and diminishes the need to rely on dominant sectors. As sectors become **more self-reliant**, this **balanced distribution of resources** promotes **better engagement**, **stronger collaboration**, and **more equitable decision-making**. This dynamic reduces the **influence of traditional power hierarchies** in intersectoral interventions and creates a **more egalitarian environment** where **mutual support** is encouraged, ultimately enhancing the **effectiveness of collaborative efforts**." | | | | |
| *PT 3 Simplified* | *Refined Context-Mechanism-Outcome (CMO) Statement  In hierarchical, low-resource settings where intersectoral collaboration is hindered by asymmetrical resource allocation and dependency dynamics (Context), the intentional redistribution of resources, through decentralised control, cross-sectoral pooling, and participatory prioritisation (Mechanisms), reduces power imbalances, fosters sectoral autonomy, and cultivates mutual accountability, leading to more equitable decision-making, sustained collaboration, and contextually adaptive interventions (Outcomes).* | | | | |
| Policy Implication | To operationalise PT3:  Mandate Cross-Sector Resource Audits:  Annual reviews comparing allocations (e.g., health vs. livelihood budgets) and redistributing surpluses.  Create Untied Fund Pools:  Designate 10-15% of sectoral budgets for joint initiatives (e.g., nutrition-sensitive livelihood programmes).  Institutionalise Shared Platforms:  Legally require sectors to co-host community meetings (e.g., Participant 3’s women-centric campaigns). | | | | |

| PT 4 | When stakeholders in settings with hierarchical structures perceive that other sectors' perspectives are not respected or lack trust in information provided by other sectors, then they withhold their input during intersectoral meetings or planning sessions. This lack of open communication impedes participation, coordination, and collaboration among stakeholders, ultimately resulting in poor engagement, poor information sharing, and limited collaborative action in programmes. | | | | |
| --- | --- | --- | --- | --- | --- |
| CMOC No | Context | Mechanism | | Outcome | Supporting quote |
|  |  | Resource | Reasoning |  |  |
| 33 | In hierarchical settings, certain sectors, like the health department, dominate discussions in intersectoral meetings. | The **authority and influence** of more powerful sectors shape the agenda and dominate decision-making. | Stakeholders from less powerful sectors feel **ignored and undervalued**, leading them to withhold their input. | Poor **engagement** and **limited participation**, reducing the effectiveness of collaborative actions. | **"The senior officer from the health department takes the floor more often, wielding a wealth of information that subtly commands attention. His words carry weight, and, more often than not, it’s his points that make it into the official record."** (Participant 14) |
| 34 | Stakeholders perceive a lack of clear agenda or inclusive representation in convergence meetings. | The absence of **structured agendas** that incorporate all departments’ indicators creates confusion. | Stakeholders feel **excluded and less motivated** to engage meaningfully in the discussion. | Weak **coordination and collaboration** among sectors during intersectoral meetings. | **"In convergence meetings, the topics of discussions should be fixed, incorporating criteria from all the departments. All departments should be aware of each other’s indicators; if this happens from the state level, it would be much better."** (Participant 5) |
| 35 | Current meeting structures fail to ensure fair participation or represent underrepresented groups like women members | Lack of mechanisms for **equal representation** and **inclusion of community voices** weakens engagement. | Stakeholders feel **disempowered**, with limited authority to push for meaningful changes in decisions. | Inequitable **representation and participation** lead to poor trust and diminished collaboration. | **"There should be fair participation from all the sectors. Meetings should also involve women members of the community, not just public representative members."** (Participant 16) |
| 36 | Public representatives feel constrained by the lack of authority in the existing framework of intersectoral meetings. | The hierarchical structure limits **decision-making power** of less influential stakeholders. | Stakeholders feel their **contributions are tokenistic**, reducing their willingness to engage further. | Reduced **trust and openness**, hampering effective collaboration. | **"As a public representative member, I don’t have much authority within the existing framework, I can only push things up to some extent."** (Participant 16) |
| 37 | Meetings do not encourage open communication or mutual respect among sectors. | The dominance of powerful sectors discourages **two-way communication** and creates a one-sided dynamic. | Stakeholders feel **hesitant to express their views**, leading to a lack of diverse perspectives in decisions. | Poor **information sharing**, with narrow and sector-biased decision-making. | **"In meetings, it’s a cacophony of voices, everyone talks, but few truly listen... The health department’s influence looms large, shaping decisions in its favour while others struggle to break through the noise."** (Participant 14) |
| 38 | Roles and responsibilities in intersectoral programmes are not clearly defined in policy documents. | Absence of comprehensive organisational guidelines outlining tasks and scope for each sector. | Ambiguity in roles allows dominant sectors to impose their priorities, sidelining less resourceful sectors. | Increased frustration and disengagement from less powerful sectors, leading to weaker collaboration. | *It’s complete chaos most of the time. We’re called to meetings and asked to ‘collaborate,’ but no one tells us what we’re actually supposed to do. Health just swoops in, takes charge, and leaves us scrambling to figure out how to contribute. Last month, during the nutrition drive, we sat there waiting for instructions, but by the time we were looped in, the health team had already made all the decisions. It’s like we’re extras in a movie where health plays the lead role, and honestly, it’s getting exhausting."* **– Participant 6** |
| 39 | More powerful sectors use their influence to assign additional tasks to less resourced sectors. | Authority and resource disparities that favour larger sectors, with little oversight of task delegation. | Arbitrary task assignments overwhelm smaller sectors, creating resentment and inefficiency. | Participants in weaker positions feel overburdened and undervalued, reducing their ability to collaborate effectively. | *"ICDS is often given extra responsibilities, like community awareness campaigns, without any additional support. It’s unfair because it’s not even part of our main role." (Participant 3, Social Welfare Officer, Dibrugarh)* |
| 40 | Conflicting directives from multiple levels of leadership exacerbate confusion at the implementation level. | Lack of coordination between district and block leadership results in conflicting messages to programme teams. | Confusion in directives leads to miscommunication and inefficiencies in programme execution. | Stakeholders feel overwhelmed and struggle to align their efforts, reducing the overall effectiveness of programmes. | *"It’s like being caught between two bosses who can’t agree on what they want. The district tells us to focus on one thing, like water supply in schools, and then the block officer says to prioritise sanitation for households. Which one are we supposed to follow? By the time we figure it out, weeks have passed, and nothing actually gets done. It’s so frustrating, how can we make progress when we’re pulled in two different directions?"* **– Participant 12** |
| 41 | Hierarchical structures create opportunities for dominant sectors to exploit unclear accountability mechanisms. | Power imbalances allow sectors with more resources to deflect responsibilities onto weaker sectors. | Lack of clear accountability mechanisms enables larger sectors to avoid responsibilities, shifting the burden. | Reduced trust and increased conflicts among sectors, undermining effective collaboration. | *"When something doesn’t work, the blame often falls on smaller departments like ours. It feels like we’re always the scapegoat." (Participant 7, Community Representative, Dibrugarh)* |
| 42 | Unclear communication channels lead to confusion about reporting structures and decision-making authority. | Lack of formalised communication protocols and reporting frameworks across sectors. | Confusion about who to report to or seek approvals from creates delays and inefficiencies in task execution. | Stakeholders feel frustrated and demotivated, further weakening intersectoral collaboration. | *"We are often told to submit reports, but it’s unclear who needs them or how they are being used. It just adds to the confusion." (Participant 8, Nutrition Officer, Dibrugarh)* |
| 43 | Meetings do not encourage open communication or mutual respect among sectors. | The dominance of powerful sectors discourages **two-way communication** and creates a one-sided dynamic. | Stakeholders feel **hesitant to express their views**, leading to a lack of diverse perspectives in decisions. | Poor **information sharing**, with narrow and sector-biased decision-making. | **"In meetings,, everyone talks, but few truly listen... The health department’s influence looms large, shaping decisions in its favour while others struggle to break through the noise."** *(*Participant 14*)* |
| 44 | Current meeting structures fail to ensure fair participation or represent underrepresented groups like women members | Lack of mechanisms for **equal representation** and **inclusion of community voices** weakens engagement. | Stakeholders feel **disempowered**, with limited authority to push for meaningful changes in decisions. | Inequitable **representation and participation** lead to poor trust and diminished collaboration. | **"There should be fair participation from all the sectors. Meetings should also involve women members of the community, not just public representative members."** *(*Participant 16*)* |
| 45 | Stakeholders engage in informal conversations to discuss unresolved or unaddressed issues from formal meetings. | Informal settings allow for **spontaneous problem-solving and idea exchange** outside structured meetings. | Stakeholders feel **enabled to share ideas and resolve issues informally**, fostering more effective collaboration. | Enhanced **teamwork and programme alignment** due to shared understanding and informal coordination. | **"We do meet often over tea and discuss a lot many things which we don’t discuss in meetings, since we know each other well it’s easier to work together more closely."** *(*Participant 14*)* |
| Thought Process | **Expanding the Context: From General Observations to Specific Challenges**  The quotes highlighted several challenges within intersectoral meetings, including **sectoral dominance, lack of inclusion, and insufficient structure**. While the context generally points to hierarchical systems, the process required narrowing down the specific challenges that stem from this hierarchy.  For example, Participant 14 described how the health department dominated discussions and decisions, creating a sense of exclusion for other stakeholders. This observation was contextualised within **power imbalances** in hierarchical systems, where certain sectors hold more authority and resources than others. By grounding this observation in the context of intersectoral collaboration, the CMOCs identified how such dominance affects the ability of less influential stakeholders to engage meaningfully.  **Identifying Mechanisms (Resources): Leadership, Structure, and Representation**  The next step involved recognising **resources offered, or absent, within the context** that influenced stakeholder behaviours. The participant quotes pointed to the following key resource gaps:  **Structured Agendas:** Participant 5 emphasised the need for clear, inclusive criteria for discussions, revealing that the lack of agenda-setting mechanisms creates confusion.  **Fair Representation:** Participant 16’s suggestion to involve women and community representatives highlighted the absence of inclusive representation as a critical missing resource.  **Equal Authority:** Participant 14 described the health department’s dominance in decision-making, showcasing how unequal authority dynamics limit input from less powerful stakeholders.  The CMOCs integrated these observations to demonstrate how the absence of these resources negatively influences engagement and collaboration.  **Recognising Mechanisms (Reasoning): Stakeholder Reactions and Perceptions**  Understanding stakeholder perceptions, how they interpreted and reacted to the context and available resources, was key to capturing the mechanisms (reasoning) driving outcomes. The quotes provided valuable insights into how stakeholders feel when excluded:  **Disempowered and Tokenistic:** Participant 16 expressed frustration about having limited authority, which reduced their willingness to engage fully in meetings.  **Ignored and Devalued:** Participant 14 noted how the dominance of powerful sectors made it difficult for other voices to be heard, causing stakeholders to feel undervalued and hesitant to participate.  **Excluded and Demotivated:** Participant 5 highlighted the confusion caused by a lack of structured, inclusive agendas, which demotivated stakeholders from engaging actively.  These insights were used to articulate the **reasoning mechanisms**, showing how stakeholder perceptions of exclusion, disrespect, and limited authority impede participation and coordination.  **Strengthening the Outcome Links: From Behavioural Patterns to Results**  Finally, the CMOCs established clear causal links between the identified mechanisms and the observed outcomes. The participant quotes revealed patterns of **disengagement, poor communication, and limited collaboration**, which were directly linked to the contextual challenges and stakeholder reasoning:  The dominance of powerful sectors (resource) created a **one-sided dynamic** (reasoning), resulting in **narrow, sector-biased decisions** (outcome).  The lack of fair representation (resource) led stakeholders to feel **disempowered** (reasoning), contributing to **poor trust and weakened collaborative efforts** (outcome).  Insufficient structured agendas (resource) caused **confusion and exclusion** (reasoning), resulting in **weak coordination and participation** (outcome).  The CMOCs thus captured the ripple effects of these hierarchical challenges, illustrating how they culminate in **ineffective intersectoral interventions**.  **Summary**  The thought process required moving from general observations of hierarchical systems to specific, actionable insights:  **Expanding Context**: Focused on how hierarchical structures manifest in intersectoral meetings.  **Resource Identification**: Pinpointed missing elements like structured agendas, fair representation, and equal authority as key challenges.  **Stakeholder Reasoning**: Captured emotional and cognitive responses (e.g., feeling disempowered, ignored) that hinder participation.  **Outcome Strengthening**: Established direct links between these challenges and the broader issues of poor engagement, weak collaboration, and limited trust.  The refined understanding demonstrates how intersectoral collaboration can falter when hierarchical dynamics are unchecked, providing a foundation for designing solutions to foster equity, inclusion, and meaningful engagement. | | | | |
| Components added: Mechanism Elaboration: Triggers | Key Components Added to refine PT 4  PT4 specifies four sub-mechanisms derived from participant data:  Neutral Facilitation of Dialogues  *Trigger*: Appointment of non-hierarchical moderators (e.g., third-party facilitators or community leaders).  *Participant Evidence*: *“The senior officer from the health department takes the floor more often… His words carry weight, and it’s his points that make it into the official record.”* (Participant 14)  Pre-Defined Decision Criteria and Agendas  *Trigger*: Jointly agreed-upon meeting frameworks (e.g., fixed topics and cross-sector indicators).  *Participant Evidence*: *“Topics of discussions should be fixed, incorporating criteria from all departments… All departments should be aware of each other’s indicators.”* (Participant 5)  Deliberative Inclusion of Marginalised Voices  *Trigger*: Quotas or rotating leadership roles for underrepresented groups (e.g., women, community representatives).  *Participant Evidence*: *“Meetings should involve women members of the community… As a public representative member, I don’t have much authority.”* (Participant 16)  Transparent Information Validation  *Trigger*: Publicly verifiable data-sharing protocols (e.g., cross-sector audits of indicators).  *Participant Evidence*: *“If (awareness of indicators) happens from the state level, it would be much better.”* (Participant 5) | | | | |
| Boundary conditions | PT4 operates effectively only when:  Powerholders Tolerate Redistribution of Influence: Dominant sectors (e.g., health department in Participant 14’s account) agree to cede discursive control.  Enforceable Agendas Exist: Pre-defined topics and indicators are codified (e.g., Participant 5’s “fixed criteria”).  Community Representation Is Legitimised: Marginalised groups (e.g., Participant 16’s women members) have institutional backing.  Trust-Building Is Incremental: Early wins (e.g., small joint decisions) establish credibility. | | | | |
| Temporal dimension | PT4’s outcomes unfold in three phases:  Contestation Phase: Dominant sectors resist ceding discursive space (e.g., health officer monopolising dialogue).  Negotiation Phase: Structured agendas and quotas dilute hierarchies (e.g., fixed topics reduce ambiguity).  Routinisation Phase: Trust becomes institutionalised (e.g., community reps routinely shape decisions). | | | | |
| Refined PT 4 | "In settings characterised by hierarchical structures, stakeholders may be hesitant to share their perspectives openly during ISC meetings if they feel that their views are **not respected** or that their contributions are **undervalued** by other sectors. This **perceived lack of respect** or **trust** creates **psychological barriers**, leading individuals to **withdraw from discussions** or **withhold critical information**. As a result, more powerful sectors or individuals **dominate the decision-making process**, exacerbating the **hierarchical nature** of the collaboration. This mechanism leads to the creation of an **unseen power structure**, where decisions become **skewed in favour of those who hold more influence or authority**. When participants do not feel **heard** or **valued**, the existing **power imbalances** within the hierarchy are reinforced, further undermining **collaborative efforts** and preventing the **full participation** needed for effective ISC." | | | | |
| PT4 Simplified | *Refined Context-Mechanism-Outcome (CMO) Statement  “In hierarchical contexts, stakeholders may hesitate to share perspectives openly during intersectoral collaboration meetings due to a perceived lack of respect or undervaluation of contributions by other sectors. This perception fosters psychological barriers, such as fear of dismissal or distrust, which lead individuals to withhold critical information (Mechanism). As a result, dominant sectors/individuals steer decision-making, reinforcing hierarchies and skewing outcomes in favour of existing powerholders. This dynamic undermines collaboration by amplifying power imbalances and excluding vital input (Outcome).”* | | | | |
| Policy Implication | To operationalise PT4:  Mandate Neutral Facilitation:  Rotate facilitators across sectors or train community leaders in mediation (addressing Participant 14’s dominance issue).  Co-Create Cross-Sector Indicators:  Develop shared metrics (e.g., health-livelihoods nexus indicators) to anchor discussions (as Participant 5 advocates).  Institutionalise Quotas for Marginalised Groups:  Reserve 30% of meeting seats for women/community reps (responding to Participant 16’s exclusion). | | | | |

| PT 5 | When policy or guidelines lack clear role delineation, then conflicts and confusion among sectors and teams arise, leading to poor engagement, inefficiency, and reduced accountability, which in turn hinders programme implementation and impacts interpersonal relationships among stakeholders at all levels. | | | | |
| --- | --- | --- | --- | --- | --- |
| CMOC No | Context | Mechanism | | Outcome | Supporting quote |
|  |  | Resource | Reasoning |  |  |
| 46 | In settings with poorly defined roles, contractual staff feel overburdened with undefined or excessive tasks. | Lack of **role clarity** and **contractual constraints** forces staff to take on multipurpose responsibilities. | Staff feel **powerless to refuse additional tasks**, leading to exhaustion and reduced job satisfaction. | Reduced **efficiency** and poor **engagement** due to overwork and low morale. | **"Yes, there is no limit or boundary to my job. I become like a multipurpose worker in this office; there is too much of a workload. I cannot say no to extra work since I am on contractual post."** (Participant 9) |
| 47 | Staff are often assigned additional responsibilities outside their original scope of work. | Lack of **structured policies** or guidelines to define roles and responsibilities clearly. | Stakeholders experience **confusion** about their roles and tasks, making coordination and efficiency difficult. | Poor **coordination** and **reduced accountability**, leading to inefficiencies in programme implementation. | **"Initially, there was a lack of clarity regarding my role leading to some confusion. I primarily focused on data management and reporting. However, as time passed, I am dragged into so many other additional tasks."** (Participant 6) |
| 48 | Arbitrary task assignment in hierarchical settings increases resentment among field-level workers. | Tasks assigned without consultation or consideration of staff capacities at the field level. | Staff feel their input is ignored, leading to demotivation and a lack of ownership over their work. | Poor morale among field workers reduces the quality of programme implementation and collaboration. | *"Tasks are often handed down to us without asking if we have the capacity to handle them. It feels like we’re being overworked with no say in the matter." (Participant 9,)* |
| 49 | Lack of clarity in operational policies causes duplication of tasks across sectors. | Overlapping mandates and inadequate policy frameworks for defining sectoral roles and responsibilities. | Duplication of tasks creates inefficiency, as sectors repeat efforts instead of working collaboratively. | Resource wastage and reduced efficiency in programme implementation | *"It’s honestly a mess sometimes. We’ll be running a hygiene awareness campaign in schools, only to find out that public health engineering has already done the same thing a week before. No one tells us who is handling what, and we just keep overlapping efforts. It’s not just frustrating, it’s a total waste of time and resources. If we actually coordinated properly, we could achieve so much more instead of repeating the same work."* **– Participant 3** |
| 50 | In settings with poorly defined roles, contractual staff feel overburdened with undefined or excessive tasks. | Lack of **role clarity** and **contractual constraints** forces staff to take on multipurpose responsibilities. | Staff feel **powerless to refuse additional tasks**, leading to exhaustion and reduced job satisfaction. | Reduced **efficiency** and poor **engagement** due to overwork and low morale. | **"Yes, there is no limit or boundary to my job. I become like a multipurpose worker in this office; there is too much of a workload. I cannot say no to extra work since I am on contractual post."** (Participant 9) |
| 51 | Dominant sector claims credit for collaborative work. | No attribution agreements. | Resentment undermines future cooperation. | Withdrawal of effort by marginalised sectors. | *“It’s always the same story, everyone works together, but when it’s time to showcase the results, only the health department gets the credit. We put in just as much effort, sometimes even more, but when reports are written or presentations are made, our contributions are barely mentioned. It’s frustrating! Why should we keep putting in the work when we know we’ll just be sidelined in the end? If this keeps happening, people will stop bothering to collaborate at all."* **– Participant 7** |
| Thought Process | **Expanding the PT: Moving from General to Specific**  The PT broadly focused on how the absence of clearly defined roles leads to confusion and conflict, negatively impacting engagement and accountability. While this was conceptually valid, it lacked specificity in addressing **how role ambiguity manifests in hierarchical health systems** and its practical implications for stakeholders.  The revised PT builds on this by situating the issue within **hierarchical structures** where power imbalances exacerbate the effects of unclear roles. Participant quotes, such as Participant 9’s description of being overburdened as a "multipurpose worker," highlighted how contractual or less powerful staff often bear the brunt of role ambiguity. This led to the explicit recognition that **role ambiguity disproportionately impacts weaker or less resourceful sectors or individuals**.  **Key Change**: The PT explicitly situates the issue within **hierarchical systems**, highlighting how power imbalances amplify the negative effects of role ambiguity, particularly for weaker stakeholders.  **Emphasising Power Dynamics and Vulnerability**  The PT briefly acknowledged inefficiencies arising from unclear roles but did not delve into the **power dynamics** that emerge from this ambiguity. The revised PT integrates evidence from the CMOCs to demonstrate how **more powerful sectors or individuals exploit unclear roles to exert dominance**. For instance, Participant 6’s experience of being dragged into additional tasks beyond their original scope highlights how role ambiguity creates opportunities for sectors or individuals with perceived authority to assign responsibilities arbitrarily.  By incorporating this dynamic, the revised PT not only focuses on the operational inefficiencies caused by unclear roles but also addresses the **psychological and relational impacts**, such as frustration, uncertainty, and resentment, especially for less powerful stakeholders.  **Key Change**: The PT introduces **power imbalances and exploitation of role ambiguity** as a central theme, showing how this dynamic exacerbates conflicts and undermines collaboration.  **Adding Mechanisms of Resource and Reasoning**  The PT mention confusion and conflict but did not clearly articulate the **mechanisms**, the resources (or lack thereof) and the reasoning of stakeholders. The revised PT identifies:  **Resource Mechanisms**: The lack of clearly defined roles or guidelines, insufficient policies, and arbitrary task allocation act as structural deficiencies that fuel role ambiguity.  **Reasoning Mechanisms**: Stakeholders feel **frustrated, undervalued, and uncertain**, particularly when they perceive their workload as disproportionate or tasks as outside their scope. Participant 5’s quote, "We need better-defined responsibilities from the start," reflects the reasoning that **clear guidelines** could prevent unnecessary conflicts and inefficiencies.  This refined focus on mechanisms enhances the explanatory power of the PT by linking the structural gaps (resources) to the behavioural responses (reasoning) of stakeholders.  **Key Change**: The PT explicitly integrates **mechanisms of resource and reasoning**, showing how a lack of clarity leads to negative perceptions, behaviours, and outcomes.  **Strengthening Mechanism-Outcome Links**  The PT touched on outcomes like inefficiency and poor engagement but did not clearly link these outcomes to the mechanisms driving them. The revised PT makes these links explicit by showing how:  **Role ambiguity leads to confusion and conflict**, which reduces efficiency and collaboration.  **Arbitrary task allocation by more powerful actors** leads to resentment and undermines accountability.  **Weaker sectors or individuals bear disproportionate burdens**, further straining interpersonal relationships and collaboration.  For example, Participant 6’s experience of being assigned tasks beyond their scope was directly linked to inefficiencies in coordination and accountability. Similarly, Participant 9’s statement about feeling overburdened as a "multipurpose worker" highlighted how unclear roles lead to exhaustion and reduced morale.  **Key Change**: The PT clearly establishes **causal links** between role ambiguity, power imbalances, and negative outcomes, including inefficiency, diminished accountability, and strained relationships.  **Incorporating Psychological and Emotional Impacts**  The PT did not fully address the **psychological and emotional consequences** of role ambiguity, such as feelings of frustration, overwhelm, and resentment. The revised PT explicitly incorporates these elements, informed by Participant 9’s statement about being unable to refuse extra work due to their contractual position and Participant 5’s emphasis on the importance of defined roles to reduce confusion.  By addressing these impacts, the revised PT provides a more comprehensive understanding of how role ambiguity affects both the operational and emotional dimensions of collaboration.  **Key Change**: The PT incorporates the **psychological burden** of role ambiguity, such as frustration and resentment, as critical factors that undermine collaboration.  Summary  The refinement of the PT into the revised PT involved:  **Contextualising the issue** within hierarchical systems, highlighting how role ambiguity disproportionately affects weaker stakeholders.  **Emphasising power dynamics**, showing how role ambiguity enables exploitation and arbitrary task allocation by more powerful actors.  **Identifying mechanisms** (resources and reasoning) that explain how role ambiguity leads to confusion, inefficiency, and strained relationships.  **Strengthening causal links**, explicitly connecting role ambiguity to negative outcomes like diminished accountability, inefficiency, and frustration.  **Incorporating emotional impacts**, recognising the psychological toll on stakeholders, especially those in vulnerable positions.  The revised PT thus provides a nuanced and evidence-based framework for understanding how role ambiguity affects collaboration, accountability, and efficiency in hierarchical health systems. It highlights the need for clear policies and guidelines to address these challenges and foster more equitable and effective intersectoral collaboration. | | | | |
| Mechanism Elaboration: Triggers | Key Components Added to refine PT 5  PT5 specifies four sub-mechanisms derived from participant data:  Formal Role Delineation  *Trigger*: Codified role descriptions embedded in policies/contracts.  *Participant Evidence*: *“We need better-defined responsibilities from the start. If everyone knows their part, there will be less confusion and unnecessary extra work.”* (Participant 5)  Participatory Task Allocation  *Trigger*: Collaborative workshops to map roles and negotiate responsibilities.  *Participant Evidence*: *“I slowly realised the importance of continuous coordination… to keep convergence alive.”* (Participant 6)  Contractual Safeguards  *Trigger*: Legal protections against role creep (e.g., clauses limiting non-contractual tasks).  *Participant Evidence*: *“I cannot say no to extra work since I am on a contractual post.”* (Participant 9)  Accountability Frameworks  *Trigger*: Transparent monitoring systems for role adherence (e.g., audits, grievance mechanisms).  Participant Evidence: “Major work was to keep the convergence alive… collaboration with various stakeholders.” (Participant 6) | | | | |
| Boundary conditions | PT5 operates effectively only when:  Leadership Commits to Role Clarity: Senior managers prioritise codifying roles (e.g., Participant 5’s call for “better-defined responsibilities”).  Participatory Processes Are Inclusive: Marginalised sectors (e.g., contractual workers) have negotiating power.  Enforcement Mechanisms Exist: Legal/policy tools penalise role violations (e.g., breaching contractual safeguards).  Resources Support Role Mapping: Time and funding allocated for collaborative workshops. | | | | |
| Temporal dimension | PT5’s outcomes unfold in three phases:  Crisis Phase: Role ambiguity leads to overload and resentment (e.g., Participant 9’s “multipurpose worker” experience).  Negotiation Phase: Participatory processes redefine roles (e.g., Participant 6’s gradual adaptation).  Stabilisation Phase: Accountability systems institutionalise clarity (e.g., Participant 5’s ideal of “easier collaboration”). | | | | |
| Refined PT 5 | "In hierarchical health system structures where roles and responsibilities are not clearly defined within organisational policies or guidelines, confusion and conflict often arise among sectors, implementation teams and individuals. This lack of clarity enables more resourceful or larger sectors to exert power over less influential ones, leading to frustration and uncertainty among participants from weaker sectors. As a result, work inefficiency, diminished accountability, and a sense of being overwhelmed emerge, particularly for those in vulnerable positions. The ambiguity in roles also creates opportunities for power imbalances, where individuals or sectors with perceived authority arbitrarily assign tasks beyond the original scope of responsibilities, further exacerbating conflicts and creating resentment. This dynamic undermines both efficiency and effective collaboration, with those in weaker positions bearing a disproportionate burden. " | | | | |
| PT 5 Simplified | *Refined Context-Mechanism-Outcome (CMO) Statement  In hierarchical health systems with ill-defined roles/responsibilities (context), power imbalances and resource disparities (mechanisms) enable dominant sectors to impose control or assign arbitrary tasks, while weaker actors face unpredictable demands. This fuels confusion, conflict, and frustration (outcomes), resulting in inefficiency, resentment, eroded collaboration, and inequitable burdens on vulnerable participants.* | | | | |
| Policy Implication | To operationalise PT5:  Mandate Role Codification in Contracts:  Include explicit task boundaries and grievance mechanisms (addressing Participant 9’s contractual vulnerability).  Conduct Cross-Sector Role-Mapping Exercises:  Co-create responsibility matrices during onboarding (as Participant 6’s adaptation suggests).  Establish Independent Oversight Bodies:  Monitor task allocation to prevent exploitation of weaker sectors (e.g., Participant 5’s “unnecessary extra work”). | | | | |

| PT 6 | When stakeholders connect beyond their professional boundaries, ISC improves. These personal connections build trust and mutual respect, making stakeholders more willing to share resources and support each other. This helps balance power dynamics and leads to better teamwork and programme success. | | | | |
| --- | --- | --- | --- | --- | --- |
| CMOC No | Context | Mechanism | | Outcome | Supporting quote |
|  |  | Resource | Reasoning |  |  |
| 52 | Stakeholders build personal relationships outside formal settings (e.g., over tea at home). | Informal **social interactions** provide opportunities for open dialogue and collaboration. | Stakeholders feel **more comfortable** discussing sensitive or complex issues in informal settings. | Improved **trust, mutual respect**, and closer collaboration among stakeholders. | **"His house is nearer to mine, and we do meet often over tea and discuss a lot many things which we don’t discuss in meetings. Since we know each other well, it’s easier to work together more closely."** (Participant 14) |
| 53 | Stakeholders engage in informal conversations to discuss unresolved or unaddressed issues from formal meetings. | Informal settings allow for **spontaneous problem-solving and idea exchange** outside structured meetings. | Stakeholders feel **enabled to share ideas and resolve issues informally**, fostering more effective collaboration. | Enhanced **teamwork and programme alignment** due to shared understanding and informal coordination. | **"We do meet often over tea and discuss a lot many things which we don’t discuss in meetings, since we know each other well it’s easier to work together more closely."** (Participant 14) |
| 54 | Informal platforms, such as smaller group discussions, are used to encourage shy stakeholders to share ideas. | Breakout sessions and informal platforms like WhatsApp for sharing thoughts outside formal meetings. | Smaller, less intimidating settings help stakeholders overcome hesitancy and share ideas more freely. | Increased participation and engagement, ensuring all sectors contribute to ISC discussions. | *"In those big meetings, it’s hard to speak up, there are so many officials, and you feel like your ideas might not be taken seriously. But during the smaller group discussions, it’s different. Last time, I talked about the need for better sanitation in the village schools, and everyone listened. It’s easier to open up in a smaller setting where you don’t feel judged or overshadowed." – Participant 13* |
| 55 | Informal gatherings, such as tea breaks or community events, allow participants to connect beyond professional boundaries. | Opportunities for informal interactions outside formal meeting settings. | Personal connections foster mutual respect and reduce hierarchical barriers between sectors. | Stakeholders feel more comfortable collaborating, leading to more cohesive teamwork and resource-sharing. | *"After one of our meetings, we all stayed back for tea, and that’s when the real conversations started. I was talking to someone from health, and we realised that our programmes for school health check-ups weren’t aligned. We ended up brainstorming right there over tea and came up with a plan to coordinate better. It’s moments like these, outside the formal setting, that make us feel like a team instead of just separate departments."* **– Participant 9** |
| 56 | Collaboration occurs spontaneously based on personal requests rather than formal structures. | Informal **mutual support and flexibility** allow stakeholders to respond to immediate needs. | Stakeholders feel **empowered to act quickly** and **proactively**, without waiting for bureaucratic approvals. | Improved **programme responsiveness** and strengthened interpersonal relationships. | **"Recently I facilitated a session on managing underweight children at a community event without formal invitation just based on the personal request. This spontaneous action was well-received, showcasing the flexibility and mutual support among colleagues."** (Participant 13) |
| 57 | Stakeholders appreciate flexibility in addressing programme needs through informal collaboration. | Informal collaboration builds a sense of **shared purpose and responsibility** among stakeholders. | Stakeholders feel **motivated and valued**, strengthening commitment to collaborative efforts. | Enhanced **mutual support, trust**, and **programme outcomes** through flexible actions. | **"This spontaneous action was well-received, showcasing the flexibility and mutual support among colleagues."** (Participant 13) |
| 58 | Informal gatherings, such as tea breaks or community events, allow participants to connect beyond professional boundaries. | Opportunities for informal interactions outside formal meeting settings. | Personal connections foster mutual respect and reduce hierarchical barriers between sectors. | Stakeholders feel more comfortable collaborating, leading to more cohesive teamwork and resource-sharing. | *"After one of our meetings, we all stayed back for tea, and that’s when the real conversations started. I was talking to someone from health, and we realised that our programmes for school health check-ups weren’t aligned. We ended up brainstorming right there over tea and came up with a plan to coordinate better. It’s moments like these, outside the formal setting, that make us feel like a team instead of just separate departments."* **– Participant 9** |
| 59 | Participants use informal communication channels, such as WhatsApp, to maintain regular and candid interactions. | Accessible and user-friendly communication platforms for day-to-day updates and support. | Informal channels enable open, unfiltered communication, strengthening interpersonal relationships. | Improved trust and camaraderie among sectors, leading to quicker decision-making and problem resolution. | *"WhatsApp is best for us. Whenever there’s an issue, like delays in mid-day meal deliveries, I can immediately message the ICDS or health team. Instead of waiting for formal meetings, we sort things out right away. It’s also helped build better relationships, when you’re constantly in touch, you start seeing each other as teammates rather than just officials from different sectors."* **– Participant 8**  *If there’s a sanitation issue or a nutrition supply delay, I just send a quick message to the officer concerned, and it gets resolved faster than it ever did before. It’s so much easier than formal letters or meetings. And over time, these small interactions have made us trust each other more, it feels like we’re all working toward the same goal."* **– Participant 12** |
| 60 | Personal connections foster resource-sharing between departments without relying on formal approvals. | Flexibility in using resources facilitated by trust and goodwill between participants. | Mutual trust and informal relationships reduce bureaucratic delays and encourage a more spontaneous exchange. | Sectors work together seamlessly, enhancing the efficiency and effectiveness of health interventions | *"We recently shared some untied funds with social welfare because they had a pressing need. It wasn’t formalised, but we trusted that they’d use it well, and they did." (Participant 4, Nutrition Officer, Dibrugarh)* |
| 61 | Informal gatherings, such as tea breaks or community events, allow participants to connect beyond professional boundaries. | Opportunities for informal interactions outside formal meeting settings. | Personal connections foster mutual respect and reduce hierarchical barriers between sectors. | Stakeholders feel more comfortable collaborating, leading to more cohesive teamwork and resource-sharing. | *"After one of our meetings, we all stayed back for tea, and that’s when the real conversations started. I was talking to someone from health, and we realised that our programmes for school health check-ups weren’t aligned. We ended up brainstorming right there over tea and came up with a plan to coordinate better. It’s moments like these, outside the formal setting, that make us feel like a team instead of just separate departments."* **– Participant 9** |
| 62 | Collaboration occurs spontaneously based on personal requests rather than formal structures. | Informal **mutual support and flexibility** allow stakeholders to respond to immediate needs. | Stakeholders feel **empowered to act quickly** and **proactively**, without waiting for bureaucratic approvals. | Improved **programme responsiveness** and strengthened interpersonal relationships. | **"Recently I facilitated a session on managing underweight children at a community event without formal invitation just based on the personal request. This spontaneous action was well-received, showcasing the flexibility and mutual support among colleagues."** *(*Participant 13*)* |
| Thought Process | **Expanding the PT: Moving from General to Specific**  The PT broadly stated that personal connections improve ISC by building trust and mutual respect, which balances power dynamics and fosters teamwork. While this captured the essence of interpersonal relationships, it lacked specificity about **how and why these connections lead to better collaboration** in hierarchical health system settings.  The revised PT contextualises the impact of personal connections by emphasising their role in **reducing hierarchical barriers**. Participant 14’s quote about informal meetings over tea provided an example of how these relationships create an environment where stakeholders view each other as **partners rather than representatives of distinct sectors**. This reframing underscores how personal connections shift the dynamics of collaboration, breaking down rigid hierarchies and enabling more fluid interaction.  **Key Change**: The revised PT explicitly highlights how personal connections **reduce hierarchical barriers**, fostering a more egalitarian and collaborative environment.  **Emphasising Trust and Resource Sharing**  The PT mentioned that trust and mutual respect improve collaboration, but it did not fully explore how these interpersonal dynamics lead to **practical outcomes** like resource sharing, candid communication, and mutual support. The revised PT incorporates insights from Participant 13’s quote about facilitating a session without formal invitations, which demonstrates how **trust-based relationships encourage spontaneous and flexible collaboration**.  By including these dynamics, the refined PT shows that trust developed through personal connections not only improves relationships but also enables **more effective sharing of resources** and **problem-solving**, leading to practical benefits in ISC.  **Key Change**: The PT adds specific mechanisms, such as **resource sharing** and **candid communication**, to explain how trust fosters meaningful and effective collaboration.  **Incorporating Flexibility and Spontaneity**  The PT focused on the formal outcomes of interpersonal trust, such as improved teamwork and collaboration, but it did not account for the **informal, spontaneous actions** that emerge from personal connections. Participant 13’s example of responding to a personal request to conduct a session highlights how flexibility and spontaneity allow stakeholders to navigate beyond rigid formalities, responding more effectively to programme needs.  The revised PT incorporates this dimension, showing that personal connections empower stakeholders to **act with greater flexibility**, bypassing bureaucratic constraints and fostering a sense of camaraderie. This ability to move beyond formal roles strengthens collaboration and ensures **timely, adaptive responses** to challenges.  **Key Change**: The PT explicitly acknowledges the role of **flexibility and spontaneity** in navigating formal structures and improving collaboration.  **Strengthening Mechanism-Outcome Links**  The PT touched on the broader outcomes of trust and respect, but it did not clearly establish the **causal pathways** between personal connections and enhanced programme success. The revised PT draws on evidence from the CMOCs to link these interpersonal dynamics to specific outcomes:  **Trust and respect reduce hierarchical barriers**, encouraging participants to view colleagues as equals rather than representatives of distinct sectors.  **Open communication and mutual support** enable smoother coordination and alignment of goals.  **Flexibility and resource sharing** lead to more fluid collaboration and timely responses to programme needs.  Participant 14’s description of discussing issues informally over tea highlights the relational aspect of these pathways, while Participant 13’s example of spontaneous collaboration illustrates the tangible results of such relationships.  **Key Change**: The PT explicitly connects personal connections to **specific outcomes** like reduced hierarchies, fluid collaboration, and enhanced programme implementation.  **Addressing Hierarchical Barriers**  The PT briefly acknowledged that personal connections improve ISC, but it did not fully address how these relationships **challenge and reshape hierarchical dynamics**. The revised PT incorporates evidence from Participant 14’s quote, which described how informal interactions reduce the perception of hierarchy and foster a sense of **shared purpose**. By including this, the PT demonstrates that personal connections not only build trust but also **redefine professional dynamics**, enabling stakeholders to collaborate as partners rather than in roles dictated by hierarchy.  **Key Change**: The PT integrates the idea that personal connections **challenge hierarchical barriers**, creating a more egalitarian and collaborative environment.  **Summary**  The refinement of the PT involved grounding the theory in evidence from the CMOCs and participant experiences while addressing gaps in the PT. The thought process followed these steps:  **Contextualisation**: Highlighted how personal connections reduce hierarchical barriers, fostering partnerships rather than sector-specific silos.  **Emphasising Mechanisms**: Identified key mechanisms like **trust, resource sharing, candid communication, flexibility**, and **spontaneity** as central drivers of improved ISC.  **Linking Mechanisms to Outcomes**: Strengthened the causal pathways, showing how interpersonal trust leads to better teamwork, collaboration, and programme success.  **Addressing Hierarchies**: Focused on how personal connections challenge and reshape hierarchical power dynamics, enabling more egalitarian collaboration.  **Integrating Evidence**: Used participant quotes to illustrate how informal interactions foster flexibility, responsiveness, and mutual respect, leading to practical benefits in ISC.  The refined PT provides a nuanced, evidence-based understanding of how personal connections foster trust, reduce power imbalances, and promote effective collaboration, making ISC more fluid, adaptive, and successful. | | | | |
| Mechanism Elaboration: Triggers | Key Components Added to refine PT 6  PT 6 now includes four sub-mechanisms derived from participant insights: **Building Informal Social Networks**  **Trigger**: Shared personal interactions outside formal settings (e.g., social gatherings, casual tea meetings).  **Participant Evidence**: "His house is nearer to mine, and we do meet often over tea and discuss a lot many things which we don’t discuss in meetings. Since we know each other well, it’s easier to work together more closely." (Participant 14)  **Facilitating Candid Dialogue**  **Trigger**: Informal settings enable unfiltered communication and shared problem-solving.  **Participant Evidence**: "We do meet often over tea and discuss a lot many things which we don’t discuss in meetings." (Participant 14)  **Encouraging Spontaneous Actions**  **Trigger**: Personal trust incentivises actions without formal invitations or rigid protocols.  **Participant Evidence**: "I facilitated a session on managing underweight children at a community event without formal invitation, just based on the personal request." (Participant 13)  **Balancing Power Dynamics**  **Trigger**: Informal, trust-based relationships create more egalitarian spaces.  **Participant Evidence**: "Since we know each other well, it’s easier to work together more closely." (Participant 14) | | | | |
| Boundary conditions | PT6’s success hinges on several conditions:  **Openness to Informal Interactions**: Stakeholders must be willing to engage outside formal spaces (e.g., Participant 14’s tea meetings).  **Mutual Respect and Reciprocity**: Trust-based relationships require all parties to contribute equitably (e.g., Participant 13’s spontaneous facilitation).  **Freedom from Excessive Bureaucracy**: Informal connections thrive in systems where rigid protocols do not stifle adaptive behaviours.  **Shared Vision and Purpose**: Personal relationships are most effective when stakeholders share overarching programme goals. | | | | |
| Temporal dimension | PT6’s outcomes develop across three phases:  **Foundation Phase**: Informal personal connections form, often outside formal meetings (e.g., Participant 14’s tea discussions).  **Trust-Building Phase**: Informal interactions lead to greater trust, candid dialogue, and mutual respect.  **Collaboration Phase**: Personal connections facilitate spontaneous, flexible, and mutually supportive actions (e.g., Participant 13’s facilitation). | | | | |
| Refined PT 6 | “In settings where participants establish **personal connections** beyond their **formal professional roles**, an environment of **mutual trust** and **respect** is fostered. This reduces **hierarchical barriers**, allowing participants to view their colleagues as **partners** rather than representatives of distinct sectors. The **trust** developed through these **interpersonal relationships** encourages participants to **share resources** more freely, provide **mutual support**, and engage in more **open, candid communication**. These dynamics promote a sense of **camaraderie** and **shared purpose**, enabling participants to navigate beyond **rigid formalities** and act with greater **flexibility** and **spontaneity**. As a result, **teamwork improves**, **collaboration becomes more fluid**, and **intersectoral health interventions** are implemented more effectively.” | | | | |
| PT 6 Simplified | Refined Context-Mechanism-Outcome (CMO) Statement *"When stakeholders forge informal, trust-based relationships beyond professional boundaries, ISC improves. These connections, rooted in personal rapport, spontaneous collaboration, and voluntary flexibility, enable stakeholders to navigate systemic constraints, share resources, and adapt to challenges in real time. This fosters egalitarian decision-making, responsive problem-solving, and sustained commitment, enhancing both teamwork and programme impact."* | | | | |
| Policy Implication | To operationalise PT6:  **Encourage Informal Stakeholder Engagement**: Organise informal events (e.g., community tea meetings, retreats) to build relationships outside formal workspaces (inspired by Participant 14’s experience).  **Incentivise Flexibility and Spontaneity**: Create policies allowing informal, need-based collaborations (e.g., Participant 13’s community facilitation).  **Strengthen Relational Accountability**: Recognise and reward collaborative behaviours that emerge from personal trust and informal spaces.  **Mitigate Hierarchical Barriers**: Provide platforms for all levels of stakeholders to engage equitably, reducing top-down power asymmetries. | | | | |
